# Unravelling Rational Design of Molecularly Imprinted Polymer for Selective Mitragynine Isolation from Kratom: Quantum Mechanical, Molecular Dynamics, and Experimental Insights

**DOI:** 10.3390/molecules31040610

**Published:** 2026-02-10

**Authors:** Untung Gunawan, Eko Adi Prasetyanto, Pretty Falena Atmanda Kambira, Dion Notario, Erna Wulandari, Enade Perdana Istyastono, Andrea Tirta Wening, Kellie Irlianto, Atthar Luqman Ivansyah

**Affiliations:** 1 Department of Pharmacy, School of Medicine and Health Sciences, Atma Jaya Catholic University of Indonesia, Jakarta 14440, Indonesia; untung.gunawan@atmajaya.ac.id (U.G.); pretty.falena@atmajaya.ac.id (P.F.A.K.); dion.notario@atmajaya.ac.id (D.N.); erna.wulandari@atmajaya.ac.id (E.W.); enade.istyastono@atmajaya.ac.id (E.P.I.);; 2 Research Center for Cheminformatics and Molecular Modeling, Atma Jaya Catholic University of Indonesia, Jakarta 14440, Indonesia; 3 The Indonesian Center for Drugs Research (ICDR), Atma Jaya Catholic University of Indonesia, Jakarta 14440, Indonesia; 4 Inorganic and Physical Chemistry Division, Department of Chemistry, Faculty of Mathematics and Natural Sciences, Institut Teknologi Bandung, Bandung 40132, Indonesia; atthar@itb.ac.id; 5 Master Program in Computational Science, Faculty of Mathematics and Natural Sciences, Institut Teknologi Bandung, Bandung 40132, Indonesia

**Keywords:** isolation, kratom, mitragynine, molecularly imprinted polymer, rational design

## Abstract

*Mitragyna speciosa*, commonly referred to as kratom, is known for its active compound, mitragynine, which is classified as a new psychoactive substance. The availability of mitragynine standards remains a challenge, highlighting the need for effective and efficient methods for isolating this compound from kratom. This study aimed to computationally design a molecularly imprinted polymer (MIP) for the selective isolation of mitragynine. Computational studies were conducted using the B3LYP def2TZVP method with DFT-D4 dispersion, and the results were verified by a laboratory experiment and a molecular dynamics study. The study revealed that methacrylic acid was the optimal monomer for MIP interactions in methanol. Laboratory experiments, employing the association constant and Job plot methods, confirmed that methanol was the ideal solvent for the pre-polymerization complex, with an equilibrium template-to-monomer ratio of 1:3. Radial distribution function analysis from molecular dynamics simulations further supported that the 1:3 template-to-monomer ratio was optimal, aligning with experimental findings. This study’s findings suggest that computational analysis may be employed for the rational design of improved MIPs and for further laboratory investigation into the selective isolation of mitragynine from plants.

## 1. Introduction

Secondary metabolites are substances generated by synthetic pathways derived from primary metabolites or that utilize substrates associated with primary metabolites. Secondary metabolites synthesized by plants that may serve as drug candidates usually include derivatives of alkaloids, phenolics, tannins, flavonoids, glycosides, terpenoids, steroids, saponins, and coumarins [1]. Standard procedures in obtaining single metabolite chemicals from plants are extraction, separation, isolation, and purification. Extraction is the process by which most inert elements remain unaltered while active chemicals in plants dissolve in solvents [2]. Extraction is a crucial initial step in the investigation of herbal plants, as key secondary metabolites must be isolated from plant components for subsequent separation and characterization [3]. Several factors that influence plant extraction include the size, texture, and water content of the plant material; the type of solvent; pressure, temperature, and extraction time; the compounds targeted for isolation; and the extraction method used [4]. Extraction methods can be divided into conventional and non-conventional methods. Conventional methods include Soxhlet extraction, infusion, maceration, decoction, reflux, percolation, and distillation. Commonly used non-conventional methods include ultrasound-assisted extraction (UAE), microwave-assisted extraction (MAE), pulse electric field extraction (PEF), enzyme-assisted extraction (EAE), supercritical fluid extraction (SFE), and accelerated solvent extraction (ASE) [5,6].

Secondary metabolites obtained during the extraction process require separation. Phytochemical separation involves isolating and purifying chemicals from plant extracts or active constituents into specific individual compounds by physical and chemical methods. The extraction of secondary metabolites has many challenges that must be overcome. The challenges include the matrix’s complexity and conformation, as well as the presence of analogous chemicals, which complicate their differentiation. Various separation techniques, including paper chromatography, column chromatography, adsorption chromatography, partition chromatography, size-exclusion chromatography, ion-exchange chromatography, thin-layer chromatography, gas chromatography, high-performance liquid chromatography, and high-performance thin-layer chromatography, can be employed to isolate and purify bioactive compounds from plant extracts [7,8]. With advancements in technology for isolating secondary metabolites from plants, there is a need for a method that selectively extracts target isolates. Furthermore, a green chemistry approach is required to enhance efficiency, reduce costs, and minimize the excessive use of chemicals [9]. A method presently under attention for isolating secondary metabolites is molecularly imprinted polymer (MIP). MIP is remarkable due to its extraordinary efficacy and specificity in extracting plant target compounds. MIP is a polymer produced by molecular imprinting, featuring unique recognition sites that are equivalent to the template in terms of shape, size, and binding groups [10].

*Mitragyna speciosa*, commonly known as kratom, is a tropical plant belonging to the Rubiaceae family. Kratom, also known as purik leaves, is indigenous to Southeast Asia, including regions such as Thailand, Indonesia, Malaysia, Myanmar, and the Philippines [11]. In Indonesia, kratom is found mainly on the island of Kalimantan, more precisely in Kapuas Hulu Regency, West Kalimantan [12]. This plant is a traditional species with numerous benefits due to its chemical components. Kratom plants possess pharmacological advantages, including analgesic-antipyretic, antibacterial, antidiarrheal, antidepressant, antioxidant, antinociceptive, and anti-inflammatory properties [13]. The constituents of kratom, particularly mitragynine and 7-hydroxymitragynine as secondary metabolites, exhibit anti-inflammatory, antidepressant, analgesic, psychotropic, and opioid characteristics, resulting in a stimulating impact at low dosages and a sedative effect at elevated concentrations. The mitragynine component is present in fresh leaves at around 38.7%, while the 7-hydroxymitragynine compound is detected at about 2% [14]. Mitragynine derivative compounds have potential for further development; nevertheless, raw materials are required as lead compounds for synthesis, reference standards, and laboratory testing. On the other hand, the raw materials for the study remain challenging to obtain in the market [15,16]. Therefore, the key objective of this research is to establish computational MIP design as an effective and efficient method for isolating mitragynine from kratom plants. This research can lower the cost of optimizing polymerization in MIP synthesis by rational design. Furthermore, it gives data and support for future experimental laboratory research. At present, no research has been conducted on the isolation of secondary metabolites in kratom using MIP, hence presenting an opportunity for research novelty.

## 2. Results

### 2.1. Computational Study

#### 2.1.1. Complex Formation and Analysis

**Figure 1:**
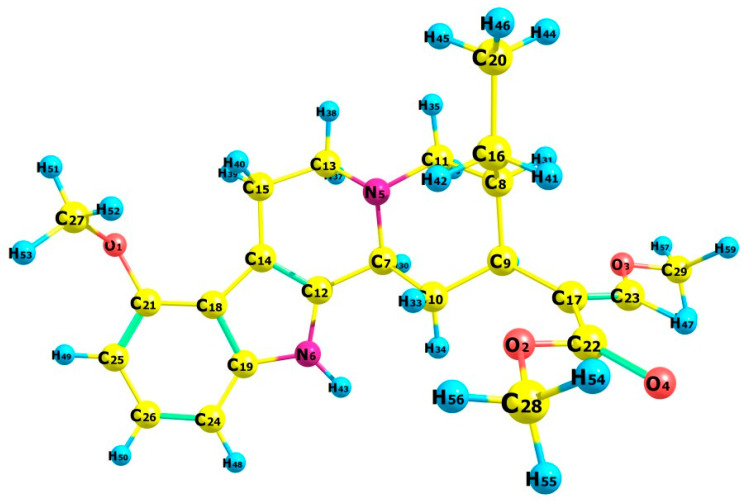
Mitragynine Structure.

**Figure 2:**
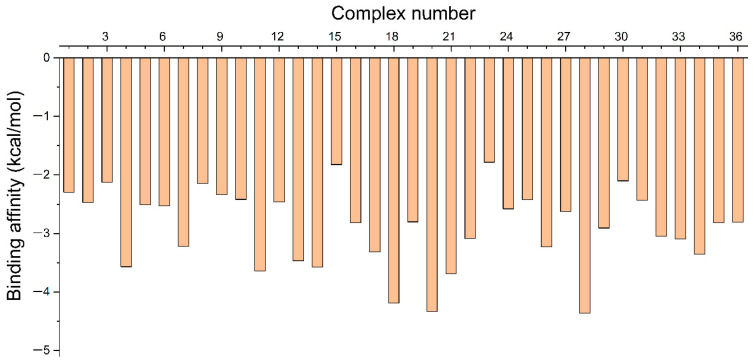
Binding affinity of the mitragynine–monomer complex.

A computational study to design a MIP for the selective isolation of mitragynine was carried out by investigating the interaction of mitragynine with various functional monomers, as illustrated in Table S1 of Supplementary Materials. The calculation was carried out using quantum mechanics employing the DFT method with the B3LYP/def2-TZVP functional and D4 dispersion correction. The robustness and suitability of the employed theoretical model were verified by comparing the experimental crystal structure of mitragynine [17] with the structure of mitragynine that was derived from computational calculations (Figure 1). This comparison served as an assessment of structural parameterization. There were no significant differences between the experimental findings and the computational modeling of geometric parameters, as shown in Table 1. Therefore, host–guest interactions involving mitragynine as a template molecule and functional monomers could be investigated at these theoretical levels. The optimized structure of mitragynine and its functional monomers were used for the next study. Molecular docking simulations of mitragynine and functional monomers were employed to obtain host–guest molecule inclusion complexes. The binding affinity and potential intermolecular interactions of each complex were assessed. Figure 2 shows that each complex’s binding affinity was negative, with the intermolecular interaction primarily driven by hydrogen and hydrophobic bonds (Table S2 of Supplementary Materials).

#### 2.1.2. Complexation Energy and Thermodynamic Study

**Figure 3:**
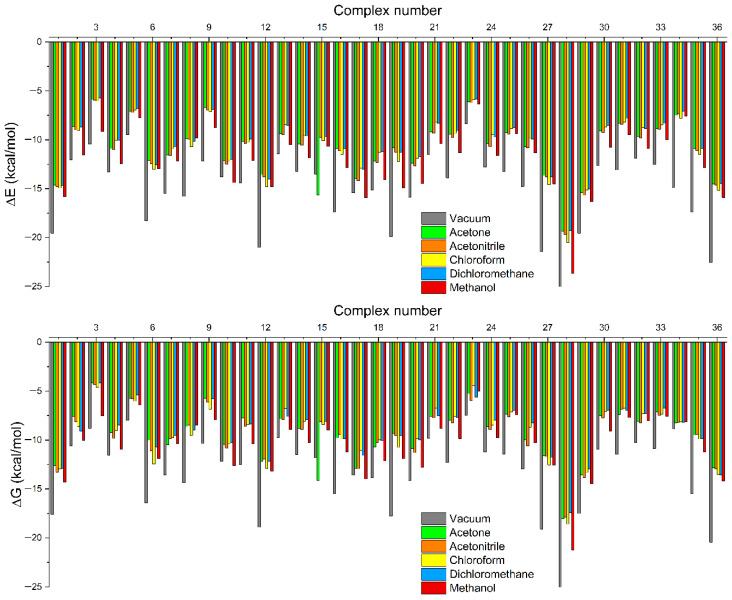
∆E_complex_ and ∆G_complex_ in vacuum and solvated conditions.

The conformations of 36 complexes with the lowest binding affinity, obtained from each molecular docking simulation, were used as the host–guest complex system for subsequent geometry optimization and frequency calculations. Figure 3 demonstrated that the lowest ∆E_complex_ value was observed under vacuum conditions. In contrast, the value varied, signifying that the solvent affects the intermolecular interactions within the complex. The thermodynamic investigation revealed a negative ∆G_complex_, indicating that all monomers could bind spontaneously to mitragynine as a template molecule. Similarly, examining complexation energy, ∆Gcomplex yields the most negative results in vacuum conditions.

#### 2.1.3. Analysis of Complex Stability

Frontier molecular orbital (FMO) analysis based on quantum–chemical parameters was conducted to assess the stability of the complex under solvated conditions [18]. FMO analysis was conducted on each complex under both vacuum and solvated conditions. The evaluated parameters included: energy gap (Eg), electron affinity (EA), ionization potential (IP), hardness (η), softness (S), chemical potential (µ), electrophilicity index (ω), stabilization energy (SE), and electronegativity (X). All parameters were determined using the HOMO-LUMO energy derivation [19]. The FMO parameters for all complexes are detailed in Table S3 of the Supplementary Materials. The analysis revealed that methanol appears to be the most suitable solvent for forming complexes.

#### 2.1.4. Selection of the Optimal Complex Formation

**Figure 4:**
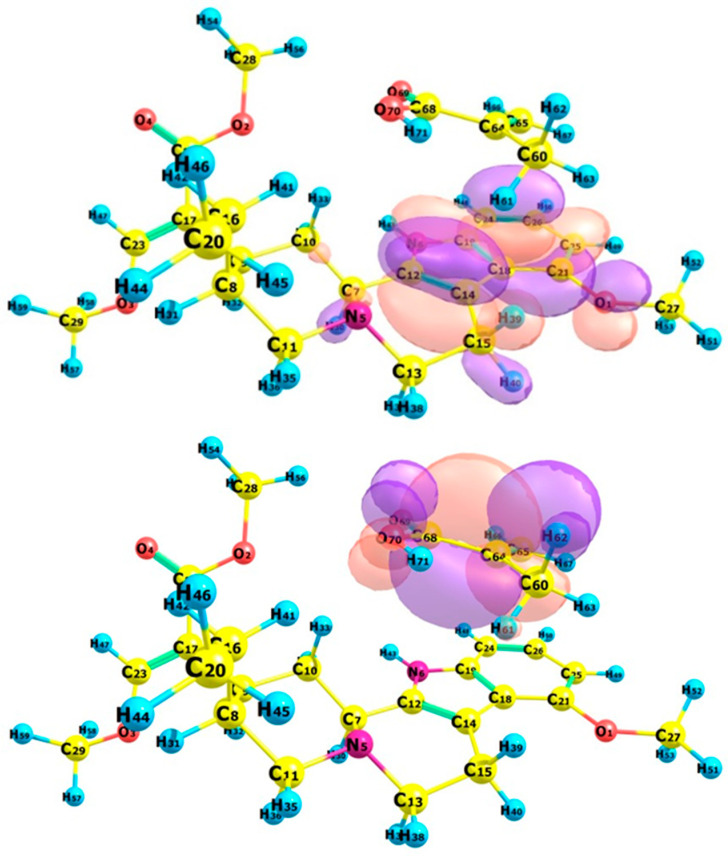
HOMO (**top**) and LUMO (**bottom**) of mitragynine–methacrylic acid complex.

Based on an integrated assessment of binding affinity, intermolecular hydrogen-bond formation, complexation energy, thermodynamic parameters, frontier molecular orbital (FMO) descriptors, and solvent compatibility, methacrylic acid (MAA) was identified as the most suitable functional monomer, while methanol was determined to be the most favorable solvent for host–guest complex formation. Figure 4 presents the HOMO–LUMO distribution of the mitragynine–MAA complex. The LUMO is predominantly localized on the host (mitragynine), whereas the HOMO is primarily localized on the guest (MAA), indicating a donor–acceptor electronic arrangement that facilitates charge transfer from the functional monomer to the template. This localization pattern is consistent with a stable pre-polymerization complex and supports the role of MAA as an effective electron donor toward mitragynine under the evaluated solvated conditions, thereby strengthening its selection as the optimal monomer for subsequent MIP design [20].

#### 2.1.5. Analysis of Non-Covalent Interactions in Complex

##### Quantum Theory of Atoms in Molecules (QTAIM)

Table S4 of Supplementary Materials reveals the topological QTAIM of complex 1 in vacuum and solvated states. A total of twelve intermolecular BCPs take place between mitragynine and methacrylic acid. The hydrogen bond in system 1, established between atom N5 in mitragynine and atom H71 from methacrylic acid, was classified as a moderate hydrogen bond, whereas 11 other systems were identified as weak hydrogen bonds, as shown by a negative value for ∇2ρ and HBCP. Additionally, the bond between N5 and H71 in system 1 had a moderate interaction strength, indicated by a |V/G| value of 1.2692, whereas the other BCPs showed weak interaction strength with |V/G| values below 1. A reliable intermolecular hydrogen bond between the nitrogen atom in mitragynine and the hydrogen atom in methacrylic acid accounts for a moderate interaction in system 1. Furthermore, the QTAIM study shows that systems 3 (H71-H42), 5 (O70-C14), and 9 (C12-O-69) only exhibit BCP in vacuum conditions. In contrast, the BCP of the systems 6 (O70-H42) and 11 (C64-C18) is not observable in vacuum states. An overview of the BCP of all complex 1 in vacuum and solvated conditions is provided in Figure S1 of Supplementary Materials.

##### Non-Covalent Interactions-Reduced Density Gradient (NCI-RDG)

**Figure 5:**
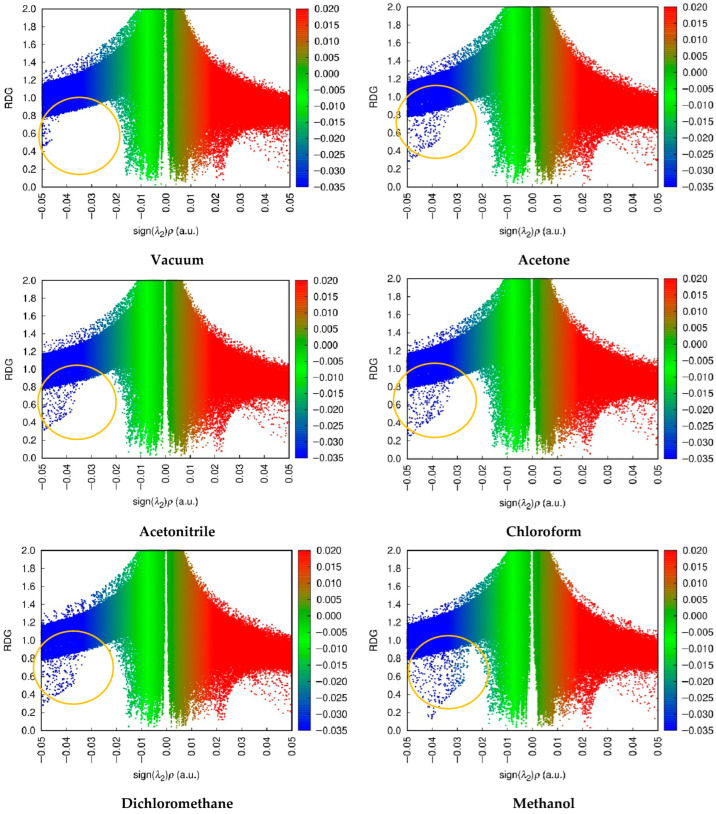
RDG scatter plot of complex 1 in vacuum and solvated conditions.

The scatter plot function of the NCI-RDG isosurface of complex 1 under both vacuum and solvated conditions is derived by multiplying electron density by the sign of the second Hessian eigenvalue (sign(λ2)ρ), which ranges from −0.05 to 0.05 a.u, as seen in Figure 5. The NCI-RDG isosurface of the complex is provided in Figure S2 of Supplementary Materials. Scatter plots of complex 1 indicated that, in contrast to the vacuum condition, solvated conditions exhibited a reduced (λ2)ρ value, signifying a strong interaction within the complex, as indicated by the yellow circle [21].

##### Interaction Region Indicator (IRI)

**Figure 6:**
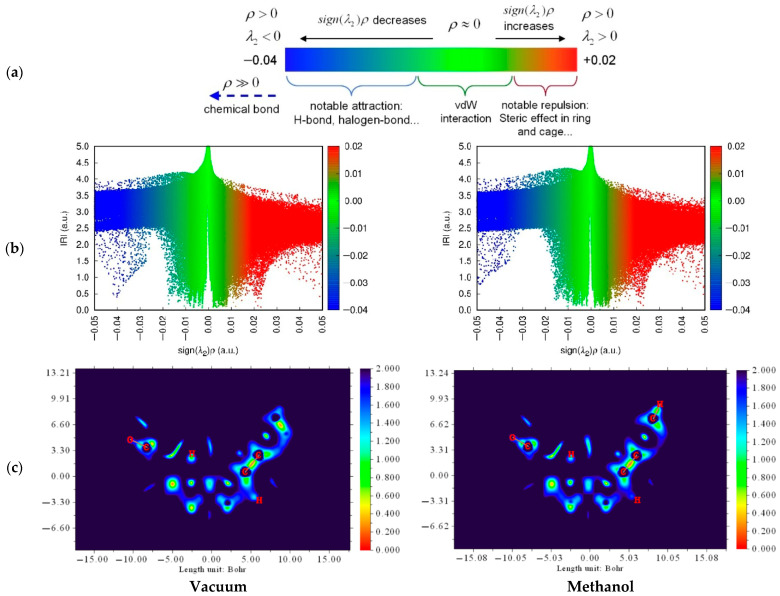
Explanation of IRI isosurface (**a**), IRI scatter (**b**), and IRI pslane map (**c**) in vacuum and methanol.

Figure 6 illustrates the IRI analysis of vacuum and methanol as the optimum solvents in prior investigations. It could be established that, from the scatter, the value of (λ2)ρ was around −0.04 under a vacuum condition. The lower (λ2)ρ value in methanol (−0.05) revealed a stronger intermolecular hydrogen bond between mitragynine and methacrylic acid.

##### Independent Gradient Model (IGM)

**Figure 7:**
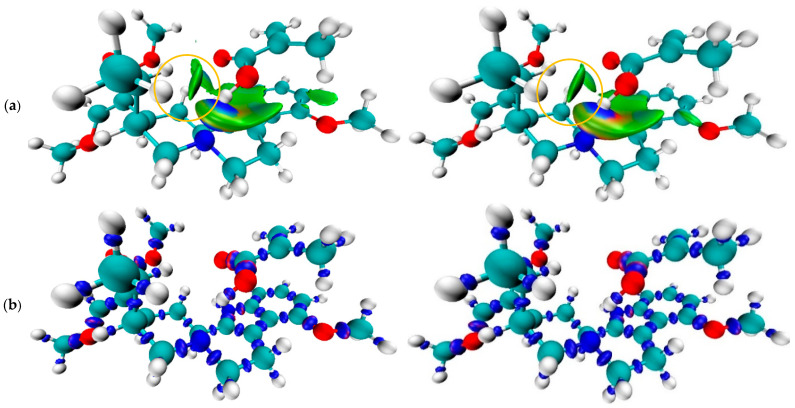

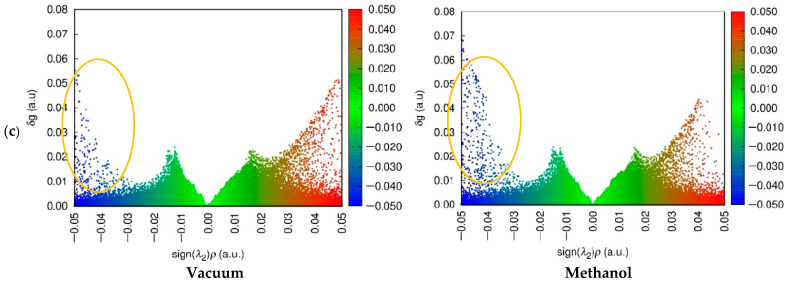
Intermolecular IGM (**a**), intramolecular IGM (**b**), and IGM scatter (**c**) in vacuum and methanol.

IGM analysis of complex 1 is illustrated in Figure 7. From the perspective of intermolecular isosurfaces, it is revealed that the interaction between the N5 atom of mitragynine and H71 of methacrylic acid, which is classified as a moderate hydrogen bond by QTAIM analysis and shown by a yellow circle (7a), exhibits differences under vacuum and methanol conditions.

##### Atomic Pair Delta G Indices (IBSIW Index)

Table 2 indicates that the main atom contributing the highest percentage in fragment 1 (mitragynine) is the N5 atom, whereas in fragment 2 (methacrylic acid), it is the H71 atom. The bond between these two atoms provides the highest atomic pair δg index in the formation of complex 1.

#### 2.1.6. Analysis of Multi-Monomer Interaction

**Figure 8:**
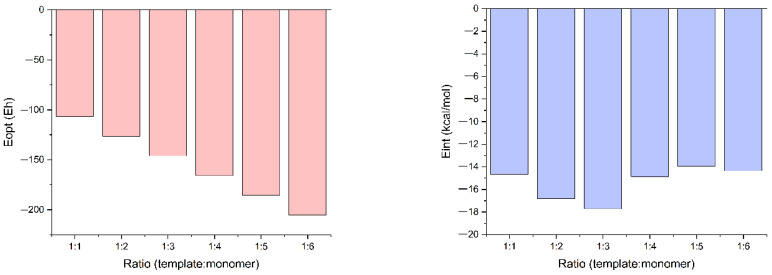
E_opt_ and E_inter_ of mitragynine–methacrylic acid complex.

Figure 8 shows that the optimization energy of the complex decreased with the increasing amount of methacrylic acid docked to mitragynine. In contrast, the interaction energy results showed that the lowest E_inter_ value was observed at a 1:3 ratio of the template–monomer complex. Representative molecular graphics illustrating the multi-monomer binding configurations of MAA around mitragynine are provided in the Figure S3 of Supplementary Materials.

### 2.2. Laboratory Study

**Figure 9:**
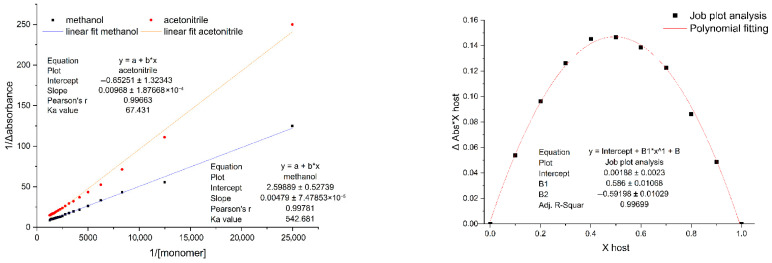
UV titration (**left**) and Job plot (**right**) of mitragynine-methacrylic acid complex.

Figure 9 shows the Ka value of the mitragynine–methacrylic acid complex in methanol and acetonitrile. Two solvents were employed to assess methanol’s performance, which was identified as the optimal solvent in the computational investigation. Acetonitrile was used as a comparison solvent, recognized for its relatively high performance, nevertheless inferior to methanol. The Job plot was investigated to determine the stoichiometry of the reaction between mitragynine as the template molecule and methacrylic acid as the monomer in the pre-polymerization complex. The absorbance was graphed as a function of the molar fraction of one component. The stoichiometry of a complex is denoted by the maximum value of the molar ratio (X host) derived from the polynomial equations [22,23]. The results demonstrate that the maximum molar ratio is achieved at x = 0.5 with a corresponding y value of 0.1465, indicating that the predominant complex at equilibrium is formed when the ratio y/x ~3.

### 2.3. Molecular Dynamics Study

#### 2.3.1. Packing System

The complex formed between T-FM from xtb docking results was used as the initial structure for subsequent complex formation with CL. During the packing process, Winmostar was used to randomly position CL molecules around the T-FM complex to form a T-FM-CL complex. This complex was then packed using methanol as the solvent to create a structure suitable for MD simulation. The formation of the packing system can be observed in Figure S4 of the Supplementary Materials.

#### 2.3.2. Molecular Dynamics Simulation

The atomic charges for the previously prepared system were assigned using the Austin Model 1–Bond Charge Correction (AM1-BCC) method. This approach was selected due to its proven reliability and computational efficiency in calculating partial atomic charges for molecular systems, especially within the framework of molecular dynamics simulations. After assigning atomic charges, the General Amber Force Field 2 (GAFF2) was utilized to define the force field parameters for all components within the system. GAFF2 was selected due to its robust and adaptable framework for modeling a diverse range of organic molecules, encompassing both small organic compounds and biomolecules. The topology (.top) and coordinate (.gro) files generated during system preparation were used as input for the subsequent MD simulations. These files define the atomic interactions, force field parameters, and initial atomic positions essential for the simulations. Following EM, the system underwent two stages of equilibration. Initially, temperature equilibration was performed under the NVT ensemble, gradually increasing the system’s temperature from 25 °C (298.15 K) to 70 °C (343.15 K). The system was then equilibrated under the NPT ensemble at 343.15 K and 1 atm for 1 ns to stabilize both pressure and density. The production MD simulation, conducted over a 10 ns period, facilitated the observation of the system’s dynamic behavior under the specified conditions.

#### 2.3.3. Refinement of MD Parameters

One of the key modifications involved increasing the number of energy minimization steps (nsteps) from 100,000 to 1,000,000. This adjustment facilitates a more thorough convergence to the potential energy minimum, ensuring a more stable initial configuration by reducing instability and unfavorable atomic interactions. Additionally, the frequency of neighbor list updates (nstlist) was increased from 10 to 20, improving the accuracy of force calculations, especially for larger systems. The cutoff distances for Coulombic (rcoulomb) and Van der Waals (rvdw) interactions were also increased from 1.0 nm to 1.2 nm, enhancing the treatment of long-range interactions, which is particularly important for systems where such interactions significantly influence the results, such as large biomolecular simulations. The energy minimization tolerance (emtol) and step size (emstep) were set to 10.0 kJ/mol and 0.01, respectively, to balance simulation accuracy and computational cost. The parameters for the NVT and NPT ensembles were further optimized to improve equilibration and ensure more stable, accurate simulations under conditions that mirror laboratory synthesis conditions. Notably, the equilibration time was extended from 1 ns to 2 ns for both NVT and NPT simulations. For the NVT (constant volume and temperature) ensembles, comparing the old and new simulation setups revealed improvements in temperature control, cutoff distances, and data output frequency. Specifically, the cutoff distances for Coulombic (rcoulomb) and Van der Waals (rvdw) interactions were increased from 1.3 nm to 1.4 nm, while the frequency of neighbor list updates (nstlist) was raised from 20 to 50. These modifications improve the accuracy of long-range interaction calculations by capturing more extensive interactions and recalculating atomic forces with greater precision. Increasing the neighbor list update frequency ensures interactions are recalculated more frequently, improving the overall accuracy of force calculations, especially in large or dynamic systems. The MD (production) phase, which is essential for observing the system’s dynamic behavior over an extended period, saw significant improvements in the new setup. The simulation time was increased from 10 ns to 40 ns.

#### 2.3.4. Analysis of MD Simulations

**Figure 10:**
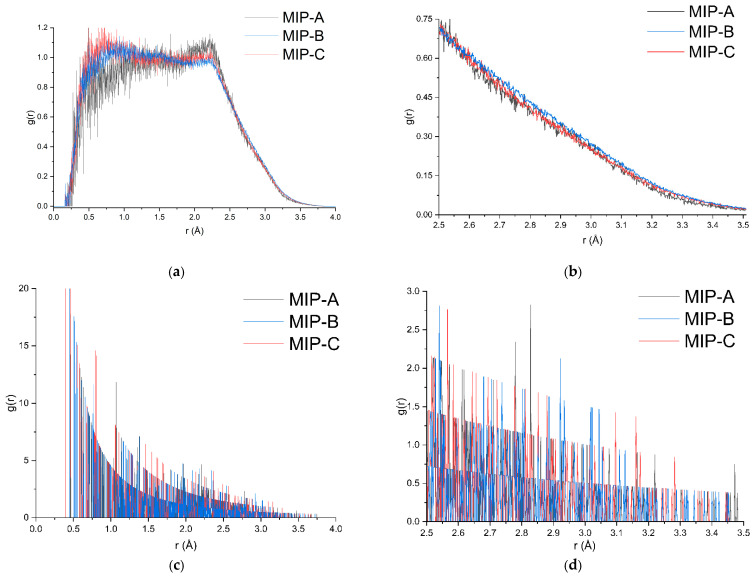
RDF profile of MIP-A, B, and C: molecule mitragynine to molecule MAA (**a**,**b**), atom N5 mitragynine and atom H71 MAA (**c**,**d**).

The post-production analysis performed using GROMACS 2025.0, xmgrace 5.1.25, Origin 10.2.5.234, and other visualization tools provided an in-depth understanding of the structural stability and dynamic behavior of the system throughout the molecular dynamics (MD) simulation. Key system properties such as potential energy, temperature, pressure, and density were evaluated to monitor the overall behavior of the system during the simulations [24]. The result of the MD post-production analysis is shown in Figure S5 of Supplementary Materials. To further investigate the system’s dynamics, the intermolecular hydrogen bonding interactions were analyzed using radial distribution function (RDF) calculations. The RDF analysis of mitragynine and methacrylic acid interactions, as shown in Figure 10, provided essential insights into the molecular interactions at different template-to-monomer ratios of 1:1, 1:3, and 1:6.

#### 2.3.5. Analysis of Crosslinker and Non-Imprinted Polymer Interactions

The molecular dynamics (MD) trajectories were post-processed to quantify interactions involving the crosslinker (CL) and to compare the non-imprinted polymer (NIP) with the corresponding MIP system under identical simulation settings. This enhanced analysis provides a more comprehensive understanding of how EGDMA affects the overall structure and fidelity of the imprinted sites, particularly considering its potential steric hindrance and weak binding interactions. Additionally, an expanded analysis of non-imprinted polymers (NIPs) was included, along with a comparison to MIPs prepared using the same system without a template molecule. This analysis was performed to isolate the specific imprinting effect and investigate the selective advantage of MAA-based MIPs.

## 3. Discussion

### 3.1. Computational Study

#### 3.1.1. Complex Formation and Analysis

The B3LYP/def2-TZVP combination in computational chemistry balances accuracy and computational cost, making it a popular choice for modeling molecular structures and properties, especially for systems where non-covalent interactions are important. B3LYP is a widely employed hybrid DFT functional, acknowledged for its effectiveness in predicting molecule structures and properties [25]. It is a hybrid functional known for its effectiveness in predicting molecular geometries. However, it may have difficulties in accurately representing non-covalent interactions, including van der Waals forces [26]. The def2-TZVP basis set offers a good balance of accuracy and computational efficiency, particularly for DFT calculations, making it a popular choice for various applications. This basis set is a triple-zeta quality basis set, extended with polarization functions, which have a higher angular momentum or diffuse functions with small exponents to introduce more flexibility, which provides a good description of electron correlation and molecular properties, especially for the host–guest interaction system [27]. The calculation was enhanced by D4 dispersion correction, which compensates for the effects of van der Waals interactions. It is an advancement over previous dispersion adjustments, such as D3, and may lead to higher system precision. The D4 dispersion adjustment markedly enhances the accuracy of calculations for systems where non-covalent interactions are critical [28,29].

A data-driven methodology was employed to categorize monomers based on their acid-base properties. Monomers 1 to 7 were classified as acidic, monomers 8 to 22 as neutral, and monomers 25 to 36 as base. This clustering approach facilitated a comprehensive examination of interactions between mitragynine and its monomers, helping identify essential characteristics that affect rational MIP design. Negative binding affinity indicates that the compound binds spontaneously without requiring energy. A lower binding affinity value indicated a more robust and stable interaction between mitragynine and functional monomers [30]. The type of intermolecular interactions within the complex was assessed, indicating that the interaction between mitragynine and most functional monomers was primarily driven by intermolecular hydrogen and hydrophobic bonds. In MIP synthesis, two primary types of interactions are commonly utilized: covalent and non-covalent interactions. The advantage of covalent interaction is that the resulting polymer has a specific bond with the template molecule, hence minimizing undesirable interactions. Nonetheless, the drawbacks include the more complicated procedure for releasing the template molecule post-synthesis, the longer duration required for rebinding, and a more limited availability of monomers. The non-covalent approach is favored for its efficient synthesis process, straightforward template removal, versatility in binding targets through monomers, and the ability to utilize a variety of monomers. The interaction comprises hydrogen bonds, dipole–dipole interactions, electrostatic interactions, and van der Waals forces [31]. The monomer capable of forming hydrogen bonds with mitragynine was preferred because further laboratory studies will focus on non-covalent interactions in MIP synthesis.

#### 3.1.2. Complexation Energy and Thermodynamic Study

The interaction between the template and the monomers influences the analyte-binding capacity of the MIP. The resultant polymers exhibited higher imprinting efficacy and a more stable complex due to stronger interactions, as evidenced by a lower complexation energy. The solvent model density (SMD) model was employed to calculate in vacuum and solvent conditions. The SMD model has become increasingly popular for the computation of condensed-phase properties and has been implemented in previous host–guest studies, among several implicit solvation models [32,33,34,35]. The investigation of complexation energy demonstrated that all complexes, both in vacuum and solvated conditions, exhibited significantly negative energy values in their ∆E_complex_ profiles, indicating an energy-driven process. The most stable complex, formed by the template and the functional monomer, is represented according to the strongest interaction (the lowest ∆E_complex_ value) [36,37]. Intermolecular interactions occur exclusively between the host and guest molecules in a vacuum. However, in solvated conditions, the possibility for interaction extends to encompass host, guest, and solvent molecules. The complex molecule exhibits an exceptional interaction with the solvent, consequently leading to a change in the intermolecular bonds, signified by a change in the ∆E_complex_ value [38]. Along with establishing robust interaction with the template, the monomer must bind to it spontaneously. Thermodynamic parameters represent the most suitable parameters for assessing the spontaneity of a process. The thermodynamic parameters were expressed in terms of Gibbs free energy (ΔG). The thermodynamic evaluation was conducted at 298.15 K and 1 atm. An exothermic reaction with a positive entropy change will invariably yield a negative change in free energy, resulting in a spontaneous reaction. The complexation process will proceed spontaneously if the Gibbs free energy is negative [39]. The solvent’s basic properties are essential to ensure the stability and spontaneity of the complexation process. Moreover, the selectivity of complexation is significantly influenced by the solvating capacity of solvents. This selectivity can substantially modify the template molecule’s binding characteristics [40].

#### 3.1.3. Analysis of Complex Stability

FMO analysis describes complex stability by analyzing the conditions of the lowest unoccupied molecular orbital (LUMO) and the highest occupied molecular orbital (HOMO). The majority of the quantum descriptors were constructed using HOMO and LUMO parameters, which were first derived from Koopman’s theorem and Parr functions within the context of DFT [41,42,43,44]. A large HOMO-LUMO gap indicates a less reactive molecule and a more stable molecule [19]. A molecule with a lower LUMO energy (more negative) possesses a stronger electron affinity, indicating it will release greater energy upon adding an electron. A higher EA value will increase the system’s stability [45]. The ionization energies of the electrons directly impact the system’s chemical reactivity and bonding properties. A higher IP value indicates an enhancement in system stability [46]. Hardness signifies a molecule’s resistance to changes in its electron distribution. A system with a higher η value is more challenging to polarize and less reactive. Hardness and softness have an inverse association [47,48]. A stable system, defined by its lack of spontaneous disintegration into smaller components, is indicated by a negative value of chemical potential [49]. A high ω value signifies a stable and electrophilic system [50,51]. Lower stabilization energy values indicate greater complex stability [52]. A higher X number indicates a decrease in reactivity, which contributes to the system’s stability [53]. The findings indicate that solvent selection markedly affects electronic interactions and the overall stability of the complexes, highlighting the significance of solvent choice in molecular complexation. The investigation suggests that methanol is the most effective solvent for forming complexes between mitragynine as the host and monomer guests.

#### 3.1.4. Selection of the Optimal Complex Formation

The selection of optimal components for MIP complex formation was based on a comprehensive assessment of several physicochemical factors characterizing interactions between mitragynine and the prospective monomers. The binding affinity and intermolecular hydrogen-bonding ability were initially assessed by molecular docking simulations to identify potential binding sites and evaluate the strength of template–monomer interactions. Complexation energy and thermodynamic parameters were computed using optimal geometries and frequency studies to assess the energetic favorability and spontaneity of complex formation. Additionally, frontier molecular orbital analysis was conducted to evaluate the electronic stability and reactivity of the resultant complexes in solvated environments, providing insights into orbital overlap, charge transfer, and solvent effects. These investigations combined offer an in-depth understanding of the compatibility of the monomers and solvents that facilitate stable host–guest complex formation. The strength and specificity of template–monomer association in MIP pre-polymerization mixtures depend on chemical complementarity between functional groups and on the solvent environment. Mitragynine is an indole-based alkaloid with base sites that can act as hydrogen-bond acceptors; therefore, stronger pre-organization is expected when acidic functional monomers provide directional hydrogen-bond donation and complementary electrostatic stabilization [54]. Consistent with this rationale, the multi-parameter screening conducted in this study identified that methacrylic acid (MAA; FM-1) in methanol as the most favorable system for host–guest complex formation. This outcome reflects not only favorable interaction strength but also consistency across independent descriptors relevant to pre-polymerization stability.

Even though MAA exhibits relatively weaker binding properties (ΔEcomplex and ΔGcomplex) values compared to others, it is important to interpret this result within the broader context of MIP synthesis, particularly considering the subsequent template removal step. While highly negative values for these parameters generally indicate strong binding, excessively strong interactions between the template and the polymer can impede the template’s detachment from the polymer matrix. This is a critical consideration, as the success of template removal directly affects the polymer’s ability to rebind the target analyte. In the case of MIPs, overly strong template–polymer interactions may hinder the efficient extraction of the template, reducing rebinding efficiency and, consequently, diminishing the overall recognition performance of the MIP [55]. Therefore, an optimal balance between binding strength and ease of template removal must be carefully considered when selecting monomers for MIP design.

The frontier molecular orbital (FMO) analysis provides additional mechanistic support for selecting the mitragynine–MAA complex. This spatial separation is characteristic of a donor–acceptor arrangement, suggesting that electron density is preferentially donated from MAA to mitragynine upon complexation. Such donor-to-template charge-transfer propensity is consistent with enhanced stabilization of the pre-polymerization complex through combined hydrogen-bonding and polarization effects, thereby favoring persistent template–monomer pre-organization in methanol [56]. Methanol was used as the solvent due to its ability to dissolve all components of the MIP synthesis and its lack of interference with hydrogen bonding between voriconazole and HEMA. A solvent parameter study demonstrated that methanol provides a stable environment for the development of the host–guest complex, thereby improving both structural integrity and interaction stability. Accordingly, the mitragynine–MAA complex (Complex 1) in methanol was selected as the optimal candidate for further characterization of its non-covalent interactions, using complementary non-covalent interaction descriptors to provide a more detailed description of the intermolecular interactions and their relevance to recognition-site reliability.

#### 3.1.5. Analysis of Non-Covalent Interactions in Complex

##### Quantum Theory of Atoms in Molecules (QTAIM)

The interaction in QTAIM is assessed by measuring the bond critical point (BCP), which corresponds to the electron density at the “saddle point” where covalent and non-covalent bonds are established between two atoms. The main parameters identified in BCP involve electron density (ρb) and its Laplacian (∇2ρb), total electron energy density (Hb), kinetic electron density (Gb), and local potential energy density (Vb) [57]. According to Rozas et al., the interaction characteristics were classified into three distinct categories based on the ∇^2^ρ and HBCP parameter: (i) Positive values of ∇2ρ and H_BCP_ indicate weak hydrogen bonds and electrostatic interactions. (ii) Negative ∇2ρ and H_BCP_ values indicate strong hydrogen bonds with a covalent character. (iii) Moderate hydrogen bonds with partial covalent characteristics are recognized when ∇^2^ρ is positive, and HBCP is negative [58,59]. The properties of bonds, specifically hydrogen bonds, are established by three criteria that Koch and Popelier proposed in accordance with the AIM theory: (i) BCP between the acceptor and donor groups confirms the presence of hydrogen bonds, (ii) ρ(r) value must be low at the BCP, dropping between 0.0020 and 0.0400 a.u., (iii) the ∇^2^ρ(r) value must be a positive, between 0.0240 and 0.1390 a.u. [60]. Moreover, the interaction strength may also be categorized using its measurement |V/G|. The weak interaction is characterized by a |V/G| value below 1, whereas moderate interaction is indicated by a |V/G| value between 1 and 2. The strong interaction occurs when the |V/G| value surpasses 2 [61].

The result indicates that, in vacuum conditions, intermolecular interactions occur only between mitragynine and methacrylic acid. However, the molecules and the solvent can interact under solvated conditions. The interactions of complex molecules with the solvent could potentially improve or weaken the intermolecular hydrogen bonds between mitragynine as the host molecule and methacrylic acid as the guest molecule. Further QTAIM analysis will be performed to assess the effect of solvated conditions, especially in system 1, as the strongest intermolecular interaction in the complex. All vacuum and solvated conditions had positive ∇2ρ and negative HBCP values, indicating the presence of moderate covalent hydrogen bonds consistently observed among all conditions. The |V/G| ratio change between solvated and vacuum conditions was identified. The enhancement in the |V/G| ratio in the solvated state signified a stronger intermolecular hydrogen bond interaction compared with the vacuum. Methanol exhibited superior performance compared to other conditions, as evidenced by the thicker blue region in the plot, indicating that the intermolecular hydrogen bonds in methanol were remarkably strong, consistent with the previous study.

##### Non-Covalent Interactions-Reduced Density Gradient (NCI-RDG)

NCI-RDG primarily emphasizes steric effects, the distribution of strong and weak interactions, and the topology of interactions in molecules. The RDG insights are presented on a scatter plot, and the interaction isosurface is illustrated. The scatter plot is divided into three sections, each represented by RGB (red-green-blue) colors. Strong interactions (sign(λ2)ρ < 0) were denoted by blue, which are classified as (i) strong intermolecular interactions, such as halogen and hydrogen bonds; (ii) intramolecular interactions, including covalent bonds and intermolecular hydrogen bonds. Weak interactions, indicated by a sign(λ2)ρ value of around 0 and represented in green, comprise two categories: (a) van der Waals forces deriving from the polarization of electron clouds and (b) hydrophobic interactions, including π-stacking. Sterical interactions (sign(λ2)ρ > 0) exhibited by red color can be identified in the cyclic structure due to ring or angle strain [62]. Methanol exhibited superior performance compared to other conditions, as evidenced by the thicker blue region in the plot, indicating that the intermolecular hydrogen bonds in methanol were remarkably strong, consistent with the previous study.

##### Interaction Region Indicator (IRI)

IRI is a significant real-space function that effectively illustrates both chemical bonds and weak interactions. IRI effectively reveals variations in electronic structure and weak interactions across an entire chemical system. The IRI function equation is analogous to the RDG function equation. The sign (λ2)1 function can be represented on IRI isosurfaces using several colors to visually illustrate the characteristics of the interaction regions identified by IRI. The symbol (λ2) represents the sign of the second-greatest eigenvalue of the Hessian of 1, which possesses a specific capacity to differentiate between attractive and repulsive interactions. On the other hand, the plane map of IRI may also illustrate chemical bonding and interactions. Regions with an IRI < 1.0 on the plane map signify weak interactions, whereas areas with an IRI > 1.0 display substantial electron density gradients or minimal electron density, making them insignificant from chemical perspectives [63]. The plane map shows an increased frequency of contact in the <1.0 region in methanol relative to vacuum, confirming the enhancement of hydrogen bonds in the solvated state.

##### Independent Gradient Model (IGM)

IGM is a powerful computational approach for visualizing and quantifying weak molecular interactions, exposing both intra- and intermolecular interactions within host–guest complexes. Under the IGM framework, the electron density gradient (∇ρ) is compared with a reference gradient (∇ρIGM), indicative of a noninteracting system. The gap between these gradients, referred to as δg, is a measure of electron sharing resulting from electron density contragradience, a concept initially developed for orbital pairs having opposite gradients. This parameter determines the electron sharing or interaction between atoms. This method offers a comprehensive, bond-specific representation of molecular interactions, enabling the examination of reaction processes and interaction atoms in the molecule [64]. The result revealed that, under vacuum conditions, the two bonded atoms are confined to the isosurface, which is dominated by blue and green colors, representing hydrogen bonds and van der Waals interactions.

In Figure 7, the interaction observed within the ring-containing region is dominated by hydrogen bonding rather than π–π stacking. The QTAIM analysis indicates that the strongest stabilizing contribution arises from a directional donor–acceptor contact between the N5 site of mitragynine and the H71 of methacrylic acid, consistent with a hydrogen-bond-driven template–monomer association. This interaction provides a localized, specific anchoring motif that governs the relative orientation of the template and monomer and is therefore expected to be a key determinant of pre-organization and recognition-site fidelity. Although π–π interactions may occur between aromatic portions of the molecules, these contacts are comparatively weaker and appear secondary to the dominant hydrogen-bond network. However, in the methanol condition, both the N5 and H71 atoms start to occupy the region inside the existing isosurface, signifying an enhancement in strength in the solvated state. Meanwhile, no difference between vacuum and solvated conditions can be observed from an intramolecular perspective. From the IGM scatter plot, the host–guest interaction at complex 1 is dominated by weak interactions, hydrogen bond (blue), van der Waals interaction (green), and steric effect (red). An analysis of the blue region in the scatter plot reveals that under vacuum conditions, the δg value approximates 0.05. However, in methanol, it is around 0.07, indicating significantly greater interactions in the solvated condition.

##### Atomic Pair Delta G Indices (IBSIW Index)

The ISBIW is utilized to evaluate the δg index of atomic pairs (δg pair), providing a quantitative indicator of the contribution of atomic pairs to the overall δg intermolecular between two fragments. This approach assesses the contribution of atomic pairs and individual atoms in interfragment interactions (atomic and atomic pair delta-g indices, also called the IBSIW index) [65]. The sum of all δg indices of atomic pairs of a given atom produces the δg index of atoms (δg atom), which represents the contribution of that atom to the interfragment interaction. The percentage of the δg index of atoms, δg atom (%), is the normalized δg atom multiplied by 100%, which can be considered a rough measure of the percentage contribution of that atom to the interfragment interaction [66]. The results reveal that the δg index increases under solvated conditions relative to vacuum, with methanol exhibiting the highest performance for mitragynine and methacrylic fragments, as well as for the formation of the host–guest complex.

#### 3.1.6. Analysis of Multi-Monomer Interaction

An additional study was conducted to assess the effect of multiple monomers in the pre-polymerization complex. In non-covalent imprinting MIP, it is crucial to determine the appropriate ratio of template to functional monomer to enhance the formation of the pre-polymerization complex and the imprinting effect. According to Le Chatelier’s principle, increasing the concentration of complex components in the pre-polymerization mixture would enhance the binding cavities in the imprinted polymer, hence increasing selectivity towards the target molecules. Matching the template’s functionality with the functional monomer is crucial, such as pairing an H-bond donor with an H-bond acceptor. The method can be employed experimentally or computationally [31]. In this study, the approach was carried out using ORCA with GFN-xTB to observe the interaction between mitragynine and multiple molecules of methacrylic acid. This method offers a balance of computational speed and reasonable accuracy, especially for small to medium-sized molecules. This method is particularly advantageous for initial docking studies or when a computationally demanding approach is not feasible [67]. GFN-xTB, a semi-empirical tight-binding method, allows for fast geometry optimizations and energy calculations, making it suitable for exploring docking poses. GFN-XTB methods are computationally much faster than higher-level quantum chemical methods or even many force fields, allowing for the exploration of a larger conformational space in a reasonable time. This method is parameterized to provide a good description of geometries, vibrational frequencies, and, importantly, for docking, non-covalent interactions, including dispersion and hydrogen bonding [68].

Mitragynine was docked with 1 to 6 molecules of methacrylic acid, corresponding to the maximum number of hydrogen bonds that it may form (1 donor and 5 acceptors). The GFN-xTB generated two primary outcomes: optimization energy (E_opt_), representing the overall energy of the optimized complex, and interaction energy (E_inter_), defined as the energy difference between the final complex with the host and guest. The final best structure with the lowest interaction energy is compared to the others. The result showed that larger systems generally have more degrees of freedom, allowing for more extensive exploration of the potential energy landscape, and larger systems are often associated with lower energy values [69]. The interaction energy results showed that the lowest Einter value was observed at a 1:3 ratio, which is the optimal ratio for forming a pre-polymerization complex between mitragynine and methacrylic acid. Figure 8 (right) indicates that this stoichiometry provides the most favorable net stabilization of the pre-polymerization complex. This behavior can be rationalized by binding-site saturation and crowding effects: up to three MAA molecules can occupy the most complementary interaction motifs on mitragynine (primarily directional hydrogen-bond donor/acceptor interaction), maximizing favorable enthalpic contributions. Beyond 1:3 (≥1:4), additional MAA molecules are forced into less optimal, more peripheral configurations where (i) template–monomer complementarity is reduced, (ii) monomer–monomer competition and steric congestion increase, and (iii) repulsive short-range contacts begin to offset any incremental dispersion or weak secondary interactions. As a result, Einter becomes less negative despite the monotonic decrease in the total optimized energy (Eopt) with system size (Figure 8 left), underscoring that Eopt largely reflects global stabilization of a larger aggregate rather than improved specific binding [70].

### 3.2. Laboratory Study

Two laboratory studies were performed to verify the prior computational method. The association constant (Ka) study analyzed the intermolecular interaction between mitragynine and methacrylic acid in the complex pre-polymerization. The Ka value was determined by the UV titration method. The Benesi-Hildebrand equation is employed by graphing 1/[monomer] [M^−1^] against 1/Δ_absorbance_ to determine the Ka value [22]. Ka values span a broad range, from extremely high (>10,000 M^−1^) to exceedingly low (<1 M^−1^). The value is less than 25 M^−1^ for weak intermolecular interactions and greater than 100 M^−1^ for strong intermolecular interactions [71,72]. The results indicate that the Ka values derived from the complex between mitragynine and methacrylic acid exceeded 100 M^−1^ in methanol, surpassing those in acetonitrile, thereby demonstrating a robust interaction between the template and the monomer in methanol, according to the computational study. In Figure 9 (left), the x-axis corresponds to 1/[monomer]; thus, the deviation from linearity at values > 10,000 reflects the dilute concentration (very low MAA concentration). Under such conditions, UV–Vis measurements are more prone to experimental and equilibrium-related influences, including minor baseline deviations, reduced signal-to-noise, and concentration-dependent variations in the apparent molar absorptivity, which may manifest as apparent non-linearity. These results indicate that future work should incorporate replicated concentration-dependent measurements and complementary validation approaches to confirm the observed behavior in the dilute regime. Job plot analysis revealed that a 1:3 ratio between mitragynine (template) and methacrylic acid (monomer) is the most favorable stoichiometry for host–guest complex formation. This observation is in strong concordance with computational multi-monomer analyses, which identified the 1:3 template-to-monomer ratio as yielding the lowest intermolecular interaction energy (E_inter_), thereby confirming its stability and suitability for molecular imprinting.

### 3.3. Molecular Dynamics Study

#### 3.3.1. Packing System

To gain a deeper understanding of potential interactions and explain the results observed in the previous study, we performed molecular dynamics (MD) simulations to explore interactions within the pre-polymerization complex. The MD simulations not only considered mitragynine as the template and methacrylic acid as the optimal monomer but also included simulations of ethylene glycol dimethacrylate (EGDMA) as the crosslinker (CL) in methanol solvent. This approach was modified from an implicit model to an explicit one. Three different ratios were used in the MD simulations, with only the mitragynine-to-monomer ratio varied to describe the effect of the template and functional monomer ratio. The EGDMA ratio was kept constant at 20, and methanol was maintained at 1000 molecules. In the MD approach, it was assumed that 1 mmol of the compound corresponds to 1 simulated molecule to simplify the system. A 20 mmol concentration of CL was chosen because, based on existing studies, it is used only as a matrix-forming agent. Its quantity must not be too low, as a rigid polymer matrix would fail to form, nor excessive, as it could reduce bonding between the template and the functional monomers. Previous studies indicated that 20 mmol yields favorable results in MIP synthesis in the laboratory [73,74,75]. T-FM ratio of 1:3, which is the optimal ratio based on the host–guest interaction studies confirmed by Job plot analysis to determine the stoichiometry, was used. The 1:1 ratio represents the minimal ratio for T-FM formation, and a 1:6 ratio represents the maximum number of hydrogen bonds that mitragynine can form, used as a comparison.

#### 3.3.2. Molecular Dynamics Simulation

The AM1-BCC method is widely adopted because it strikes a favorable balance between accuracy and computational cost, making it particularly well suited for large-scale simulations. By employing semi-empirical quantum mechanics, this method provides a more accurate depiction of electrostatic interactions than simpler charge models. Moreover, the AM1-BCC method can handle a broad range of molecular structures, including both organic compounds and biomolecules, making it an appropriate choice for the current system. Assigning precise atomic charges using this method improves the overall reliability of simulations, thereby enhancing the accuracy of predictions of molecular behavior and interactions [76,77]. GAFF2 enables efficient, reliable simulations of intricate systems. The force field effectively captures essential intramolecular and intermolecular interactions, including van der Waals forces, electrostatics, bond stretching, angle bending, and torsional deformations. Moreover, the parameters of GAFF2 are derived from a comprehensive set of quantum-mechanical calculations and experimental data, further enhancing its precision in representing molecular energetics. Consequently, the use of GAFF2 enables high-fidelity simulations of the system’s behavior, making it an appropriate choice for the current investigation [78].

EM was performed using the steepest descent algorithm to refine the system’s geometry, minimizing unfavorable atomic contacts in the initial configuration. This step was performed to carefully adjust the system’s geometry while preventing instability, thereby ensuring that the system reached a stable potential-energy minimum [79]. This controlled heating procedure in NVT was designed to ensure uniform temperature distribution throughout the system, preventing the introduction of excessive stresses that could potentially disturb the molecular structure. The temperature increase from ambient conditions to 70 °C corresponds to the actual conditions typically employed in laboratory synthesis, where the boiling point of methanol is 65 °C. Additionally, the most commonly used initiator in this context is Azobisisobutyronitrile (AIBN), which decomposes via thermolysis (with a decomposition temperature range of 50–70 °C) and photolysis. This temperature range is commonly used in the synthesis of molecularly imprinted polymers (MIPs) to facilitate polymerization [80]. The NPT ensemble allows the temperature and pressure to fluctuate, promoting the system’s adaptation to equilibrium under the specified conditions. The production phase provided valuable insights into the molecular motions, structural alterations, and potential interactions within the system, offering a comprehensive understanding of its behavior over time. A 10 ns simulation time was deemed sufficient to capture significant conformational changes and served as an initial study in MD simulations.

#### 3.3.3. Refinement of MD Parameters

The refinement of molecular dynamics (MD) parameters aimed to enhance the precision and accuracy of the simulations, ensuring they more closely reflect the conditions encountered during laboratory synthesis while maintaining computational efficiency. The refinement allows for the observation of long-term molecular motions and significant conformational changes. This extension of the simulation duration enables deeper exploration of the system’s behavior over time. Simultaneously, reducing the data output frequency minimizes file size, improving simulation efficiency while retaining the essential data needed for analysis. The use of compressed trajectory data further reduces disk space usage, an important consideration for longer simulations. A noteworthy modification in the new MD setup is the adoption of the Parrinello–Rahman method for pressure control, replacing the Berendsen method used in the previous setup. The Parrinello–Rahman method offers more accurate pressure control, particularly in molecular dynamics simulations where pressure fluctuations are significant. This improvement enhances the system’s stability during equilibration and ensures more reliable performance during the production phase [81].

#### 3.3.4. Analysis of MD Simulations

Post-production analysis revealed that the system’s potential energy remained stable over time, indicating that it had reached a thermodynamically stable state after initial equilibration. Fluctuations in potential energy were minimal, suggesting that the system’s overall configuration remained relatively constant, thus confirming the structural stability of the MIP system during the simulation period. This stability is crucial to preventing the resulting polymer network from undergoing significant conformational changes, thereby ensuring the reliability of the final imprinted structure. The temperature and pressure profiles also exhibited minimal fluctuations, signifying that the system was maintained at near-constant conditions throughout the simulation. This controlled environment is essential for studying the intrinsic behavior of the MIP system, as it prevents external variables from interfering with molecular interactions and the polymer-forming process. Additionally, the system’s density remained relatively stable, supporting the assumption that the polymer network did not undergo any significant phase transitions or volume changes that might affect the polymerization process or the final MIP structure. Furthermore, root-mean-square deviation (RMSD) and root-mean-square fluctuation (RMSF) analyses were performed to assess the system’s structural dynamics and flexibility. RMSD values were used to monitor the system’s conformational stability over time. A steady RMSD profile suggests that the system reached equilibrium after initial equilibration and that the polymer network remained stable throughout the simulation. RMSF analysis provided insights into the flexibility of individual components of the system, highlighting regions of the polymer network that may exhibit higher or lower flexibility. These regions, especially those with higher RMSF values, may play critical roles in the interaction strength between the template and the functional monomer during the molecular imprinting process [82].

In this study, RDF was employed to assess the interactions between mitragynine (the template molecule) and methacrylic acid (the functional monomer) at different ratios, specifically 1:1, 1:3, and 1:6. The RDF calculation allows for the determination of the proximity and ordering of molecules or atomic pairs, offering valuable insights into how these interactions evolve during the molecular imprinting process [83]. To understand the interaction behavior between mitragynine and methacrylic acid in the polymerization complex, RDF was used to track the spatial arrangement and interatomic distances over the course of the simulation. The analysis highlights the distances at which significant interactions, such as hydrogen bonding and electrostatic interactions, occur between the atoms or molecules. In particular, we focused on the specific atomic interactions between N5 of mitragynine and H12 of methacrylic acid, as well as the overall molecular arrangement of the system.

The RDF analysis was divided into two key distance regions: 0 to 5 Å, generated by the software, and the more specific range of 2.5 to 3.5 Å, which represents the hydrogen bond distance between mitragynine and methacrylic acid (MAA) in both RDF analyses [84,85]. The insights from the first analysis, which compared the RDFs of mitragynine and MAA (molecule-to-molecule), helped identify the key interaction regions responsible for imprint formation and the polymer’s selectivity towards the template. Variation in the template-to-monomer ratio significantly affects overall molecular interactions, providing a comprehensive understanding of the system’s behavior as a function of the mitragynine-to-methacrylic acid ratio. The RDF profiles corresponding to template-to-monomer ratios demonstrate clear distinctions in their molecular interaction patterns. Among these, MIP-B exhibited the most favorable RDF characteristics, as presented in Figure 10b, with g(r) values predominantly exceeding those observed for MIP-A and MIP-C. Notably, the average g(r) value within the critical hydrogen bonding range of 2.5–3.5 Å was highest for MIP-B (0.2975), compared to MIP-A (0.2853) and MIP-C (0.2788), further supporting the superior molecular recognition associated with the 1:3 ratio.

The second RDF analysis was performed on the N5 atom of mitragynine and the H71 atom of MAA, which are the key atoms involved in intermolecular hydrogen bonding in the previous experiments. In the N5-H71 RDF analysis, distinct, sharp peaks indicated well-defined, strong intermolecular interactions, in contrast to the molecule-to-molecule RDF analysis. The prominent peak typically corresponds to the closest molecular approach, suggesting a strong intermolecular interaction between mitragynine and methacrylic acid, likely driven by hydrogen bonding and π–π stacking. These interactions contribute to the formation of a stable and ordered polymer network, which is critical for high template retention and specific imprinting. The RDF profile suggests that MIP-B promotes a high degree of molecular organization, leading to a well-defined polymer structure that can efficiently recognize and bind the template molecule, with an average *g*(*r*) at 2.5–3.5 Å of 0.3002 compared to MIP-A (0.2944) and MIP-C (0.2895). This indicates that the 1:3 template-to-monomer ratio is the most favorable for producing MIPs with strong binding affinity and selectivity for mitragynine. The findings from this RDF analysis further reinforce the conclusion that the 1:3 template-to-monomer ratio is optimal for MIP synthesis, as it demonstrates strong, stable intermolecular interactions between the template and monomer. These interactions are critical for molecular recognition applications, such as selective binding in the MIP application. Both findings from RDF analyses align with earlier multi-monomer analyses and laboratory experiments, which further confirm the superior imprinting quality at this ratio for subsequent laboratory synthesis.

#### 3.3.5. Analysis of Crosslinker and Non-Imprinted Polymer Interactions

**Figure 11:**
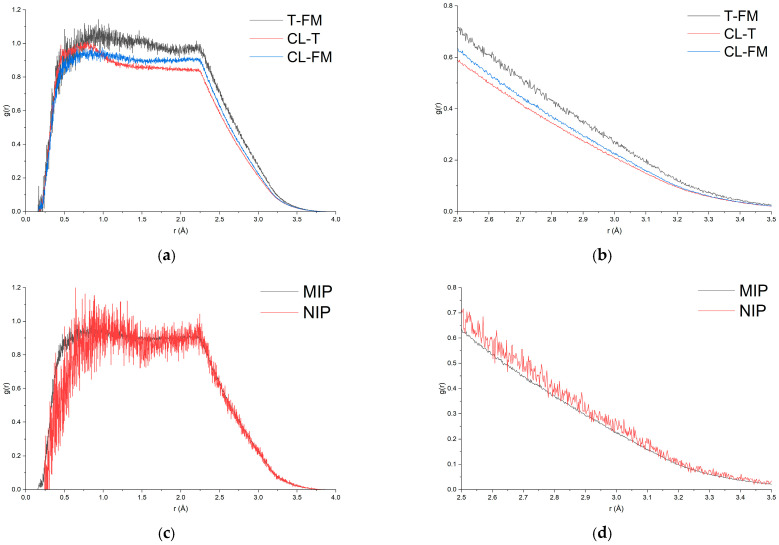
RDF profile of template (T), monomer (FM), and crosslinker (CL) (**a**,**b**); RDF interaction of CL-FM in MIP and NIP (**c**,**d**).

MD post-processing suggests that the crosslinker (EGDMA), although commonly treated as a purely structural scaffold, contributes to the local microenvironment of the pre-polymerization complex through steric constraints and weak, transient non-covalent interactions. This interpretation is supported by the distinct RDF features observed for EGDMA relative to both mitragynine and MAA in the MIP system (Figure 11a,b), indicating a non-random spatial organization of the crosslinker around the template–monomer assembly. Crosslinker–template (CL–T) and crosslinker–monomer (CL–M) contacts are evident; however, these interactions are weaker than the template–functional monomer (T–FM) interactions, as reflected by the lower RDF intensity in both the 0–4.0 Å range and, more critically, in the 2.5–3.5 Å region typically associated with hydrogen-bond formation. Consequently, EGDMA appears to play a secondary stabilizing role, whereas the dominant driving force for complex formation remains the directional template–MAA association. These findings further imply that crosslinker content should be carefully balanced, since excessive crosslinker may introduce steric crowding that perturbs optimal T–FM organization, whereas insufficient crosslinking may compromise the structural integrity and recognition sites during polymerization [84].

The NIP simulations provide a computational control to approximate the template’s contribution in the pre-polymerization mixture. In the absence of mitragynine, the structured arrangement of MAA around a template-defined region is absent, and the distribution of monomer-crosslinker contacts becomes more uniform. This is confirmed by the RDF profiles, where the probability of crosslinker–functional monomer (CL–FM) interactions in the NIP system is higher than in the MIP system, consistent with the absence of competitive template–functional monomer (T–FM) interactions in the NIP model (Figure 11c,d). Collectively, the contrasting interaction patterns between MIP and NIP support the conclusion that the template plays a significant role in MIP-mediated cavity formation. Although MD-level evidence cannot replace adsorption-based selectivity measurements, it provides mechanistic support for the imprinting hypothesis and offers practical guidance for optimizing subsequent laboratory synthesis conditions.

### 3.4. Study Limitations and Future Optimization Directions

The current study has several intrinsic limitations that should be acknowledged. First, the work primarily establishes a computational framework for rational MIP design, supported by pre-polymerization experimental validation of the optimal template–monomer complex. Full MIP synthesis and performance evaluation (adsorption isotherms, selectivity, reusability, etc.) were beyond the scope of this study. In addition, MD outcomes are limited to the pre-polymerization state, as polymer network formation and curing kinetics were not explicitly modeled; thus, translation to final morphology and site heterogeneity requires experimental verification. Second, although the computational workflow and pre-polymerization validation support the rational selection of the mitragynine–MAA system, matrix interference was not explicitly examined. In practical applications, mitragynine is typically isolated from complex kratom extracts containing structurally related alkaloids and other co-extractives that may competitively occupy recognition sites or promote non-specific adsorption. Such competition can reduce apparent binding capacity and selectivity relative to idealized single-analyte conditions, thereby attenuating performance predicted by template-focused simulations. Third, the current study does not provide data on long-term stability and operational durability. For real separation workflows, MIPs must maintain binding performance over storage and repeated adsorption–desorption cycles. Stability effects such as swelling/shrinkage, pore collapse, oxidation, or gradual site deformation can alter binding-site accessibility and reduce imprinting factor over time. Future work should integrate targeted synthesis with application-oriented evaluation, focusing on the selective isolation of mitragynine from kratom matrices under realistic conditions.

## 4. Materials and Methods

### 4.1. Computational Study

#### 4.1.1. Complex Formation and Analysis

A computational method was performed to obtain the best MIP component to interact with mitragynine as the template molecule. MarvinSketch was used to illustrate the structure of 36 functional monomers, as illustrated in Table S1 of Supplementary Materials. The computational method employed recognition of a chemical nature via host–guest interactions, with mitragynine acting as the host and monomers as guest molecules. Avogadro was used to build the 3D structure of mitragynine and monomers [85], subsequently followed by geometric optimization of all initial structures utilizing ORCA using B3LYP/def2-TZVP with D4 dispersion correction to accurately account for noncovalent interactions [86]. The default ORCA software convergence criteria were applied throughout the optimization process. The structural parameters are investigated to ensure that the computational methodologies are appropriate. Optimized molecular structures of mitragynine and monomers were utilized to generate template–monomer complexes. Molecular docking simulations were performed to generate the structure of complexes using Yasara [87]. In the molecular docking simulation, the mitragynine, serving as the host, was maintained rigid, whilst the monomers, acting as guests, were allowed to be flexible. The simulation upholds a 1:1 mole ratio between the host and guest molecules. The complexes generated from molecular docking simulations were evaluated to assess the intermolecular interactions between mitragynine and monomers using Discovery Studio Visualizer [88].

#### 4.1.2. Complexation Energy and Thermodynamic Study

The complex structures with the lowest binding affinity from each molecular docking simulation were chosen for geometry optimization and frequency calculation by calculating the minimum energy and Gibbs free energy. The examination was conducted under gas-phase (vacuum) and solvated conditions employing the solvent model density (SMD) method. The solvation was performed in acetone, acetonitrile, chloroform, dichloromethane, and methanol, which are capable of dissolving mitragynine and other MIP components and are commonly used in experimental MIP synthesis. These solvents were chosen in order to evaluate the impact of solvent polarity on complex stability and to simulate realistic laboratory conditions. The default solvent descriptors included in ORCA 6.0, especially the SMD–CDS parameter set, were used in all solvation computations. The corresponding solvent parameters, including dielectric constants and refractive indices, were assigned using internal identifiers such as Soln, Soln25, Sola, Solb, Solg, Solc, and Solh, as outlined in the ORCA manual. The calculation of complexation energy (∆E_complex_) and Gibbs free energy (∆G_complex_) is performed using the following equation:

∆E_complex_ = E_complex_ − (E_mitragynnine_ + E_monomer_) (1)

∆G_complex_ = G_complex_ − (G_mitragynnine_ + G_monomer_) (2)

#### 4.1.3. Analysis of Complex Stability

The stability of the mitragynine–monomer complex structure was assessed using the frontier molecular orbital (FMO) assessment based on the states of the highest occupied molecular orbital (HOMO) and the lowest unoccupied molecular orbital (LUMO). Chemcraft was employed to observe the structural parameters and visualize the three-dimensional structures of all complexes formed under vacuum and in solvated conditions [89]. The energy gap (Eg), electron affinity (EA), ionization potential (IP), hardness (η), softness (S), chemical potential (µ), electrophilicity index (ω), stabilization energy (SE), and electronegativity (X) parameters were determined using the HOMO-LUMO energy derivation.

E_g_ = E_LUMO_ − E_HOMO_ (3)

EA = −E_LUMO_ (4)

IP = −E_HOMO_ (5)

η = (E_LUMO_ − E_HOMO_)/2 (6)

S = 1/2η(7)

(8) $$µ=\frac{1}{2}(E_{\mathrm{HOMO}}+E_{\mathrm{LUMO}})$$

ω = µ^2^/2η (9)

#### 4.1.4. Selection of the Optimal Complex Formation

Optimal monomer and solvent candidates for host–guest complex formation were selected using a systematic computational methodology. Molecular docking simulations were used to determine the binding pose and evaluate binding affinity, providing initial insights into interaction sites and orientations, as well as into intermolecular interactions. The resultant compounds were further analyzed for complexation energy to assess their energetic stability across various solvent conditions. Thermodynamic parameters were computed to assess the spontaneity and thermodynamic stability of the complexation process. FMO research was used to investigate electronic interactions, including orbital overlap and charge-transfer properties, which reveal the compatibility of each monomer–solvent pair with the template molecule. This multi-parameter assessment facilitated a thorough comparison of the evaluated systems, ensuring that the next investigation concentrated on the most optimal template–monomer complex.

#### 4.1.5. Analysis of Non-Covalent Interactions in Complex

The interaction behavior between the template and the monomer in the complex was analyzed using various methods, including QTAIM, NCI-RDG, IRI, IGM, and IBSIW index to investigate the interactions between mitragynine as the template molecule and the optimized functional monomer for MIP synthesis that cannot be observable through experimental study in the laboratory, utilizing the multiwfn [90], VMD [91], Gnuplot [92], and the GIMP software package [93].

#### 4.1.6. Analysis of Multi-Monomer Interaction

The optimal monomer candidates from the previous study were further analyzed using GFN-xTB simulations to examine the effects of multiple monomers on mitragynine, the template molecule. The extended semiempirical tight-binding model evaluated the interactions and behavior of mitragynine with multiple monomer molecules.

### 4.2. Laboratory Study

The intermolecular interaction in the complex between mitragynine and the monomer was examined by measuring association constants (Ka) and conducting Job plot analysis using mitragynine and methacrylic acid (MAA), the most promising monomer candidate determined by computer simulation. The Ka was determined by UV titration; a methanol solution of mitragynine was added continuously to methacrylic acid, starting at 10 µg/mL, until a 20-fold excess was achieved. Absorbance measurements were carried out at the maximal wavelength of mitragynine for each increment in the functional monomer solution. A graph was constructed to illustrate the correlation between the concentration of the functional monomer and the change in absorbance (delta absorbance). Job plot analysis was conducted by varying the molar fraction of mitragynine, the template molecule, with MAA as the functional monomer. The measurement approach involved preparing a series of solutions containing mitragynine and MAA, maintaining a constant total concentration of template and monomer, and varying the [mitragynine]/[MAA] ratio at a total volume of 3 mL. The change in absorbance (ΔA) was graphed against the molar fraction of each template to assess the complex stoichiometry. The Ka and the Job plot investigations were performed in methanol, which is the most appropriate solvent from the computational study. The Benesi-Hildebrand equation was used to determine the Ka value and stoichiometry of the complex from this graph.

(10) $$\frac{1}{∆A}=\frac{1}{A∆HG}Ka\left[G\right]+\frac{1}{A∆HG}$$

∆A: absorbance change, [G]: monomer concentration (M), HG: template–monomer complex concentration (M), and Ka: association constant (M^−1^).

### 4.3. Molecular Dynamics Study

#### 4.3.1. Packing System

Molecular dynamics (MD) simulations were conducted using a molecular system intended for laboratory synthesis. The structures of mitragynine, methacrylic acid, ethylene glycol dimethacrylate (EGDMA), and methanol were used from a previously optimized structure obtained using the DFT method. For the subsequent synthesis, the component ratio of mitragynine (as the template) to methacrylic acid will be set at 1:3, based on prior optimization results. Additional components include EGDMA as the crosslinker and methanol as the solvent, which has been identified as the most suitable medium in previous studies. For comparison, simulations will also be performed using template-functional monomer (T-FM) ratios of 1:1 and 1:6. The mitragynine–MAA complex obtained from previous xtb docking results was packed using 20 molecules of crosslinker and 1000 molecules of methanol. The molecular packing of pre-polymerization components was performed using Winmostar using periodic boundary conditions (PBC).

#### 4.3.2. Molecular Dynamics Simulation

Molecular dynamics (MD) simulations were performed using the GROMACS software package [94,95]. The previously packed system was assigned atomic charges using the AM1-BCC method. The GAFF2 was employed to define the force field parameters for all components in the system. The resulting topology (.top) and coordinate (.gro) files generated during the setup were utilized as input for the MD simulations. Energy minimization (EM) was carried out using the steepest descent algorithm for up to 100,000 steps, with a convergence criterion of 100.0 kJ/mol/nm and a step size of 0.01 nm. Non-bonded interactions were calculated using a 1.0 nm cutoff, and long-range electrostatics were treated using the Particle Mesh Ewald (PME) method. The Verlet cutoff scheme and a neighbor list update every 10 steps ensured stability prior to equilibration. The system was equilibrated under the NVT ensemble from 25 °C (298.15 K) to 70 °C (343.15 K) for 1 ns using the md integrator and a 0.001 ps timestep. Temperature control was maintained via the v-rescale thermostat (τ = 0.1 ps), with periodic boundary conditions applied and center of mass motion removed. Subsequently, an NPT equilibration was conducted at 343.15 K and 1 atm for 1 ns to stabilize pressure and density, using the same integrator and timestep settings as the NVT phase. The production MD simulation was performed for 10 ns with a 0.001 ps timestep under constant temperature and pressure conditions, enabling observation of the system’s dynamic behavior.

#### 4.3.3. Refinement of MD Parameters

The refinement of molecular dynamics (MD) parameters was performed to enhance the accuracy and precision of the simulations, ensuring they more closely represent the conditions encountered during laboratory synthesis while maintaining simulation efficiency. Initially, the electrostatic minimization (EM) parameter was improved by increasing the total number of steps (nstep) to 1 million, with the energy minimization tolerance (emtol) set to 10 kJ/mol. Additionally, the parameters for the NVT (constant volume and temperature) and NPT (constant pressure and temperature) ensembles were adjusted, and the simulation time was extended from 1 ns to 2 ns. For the production run, further improvements were made, and the simulation duration was increased from 10 ns to 40 ns.

#### 4.3.4. Analysis of MD Simulations

Post-production analysis was performed using GROMACS, xmgrace, and other visualization tools. General system properties were evaluated, including potential, temperature, pressure, density, root mean square deviation (RMSD), and root mean square fluctuation (RMSF). Additionally, specific molecular interactions were analyzed using radial distribution function (RDF) calculations. These analyses provided a comprehensive understanding of the system’s structural stability and dynamic behavior throughout the simulation.

#### 4.3.5. Analysis of Crosslinker and Non-Imprinted Polymer Interactions

To investigate the role of the crosslinker (EGDMA) and its influence on the fidelity of imprinted recognition sites, the pre-polymerization complex simulations were expanded to include a detailed analysis of interactions among EGDMA, the template, and the functional monomer under identical simulation conditions. Crosslinker-specific interactions were evaluated using RDF analysis, following the same workflow described in a prior study. For assessing template interactions, thereby enabling a consistent comparison of spatial organization and interaction patterns across MIP and NIP models.

### 4.4. Chemicals, Instruments, Software

Mitragynine and methacrylic acid were purchased from Sigma Aldrich (Darmstadt, Germany), methanol and chloroform were purchased from Merck (Darmstadt, Germany), and all chemicals are analytical grade. A UV/Vis spectrophotometer, Shimadzu UV1900i (Shimadzu Corporation, Kyoto, Japan), was utilized for laboratory study. The following software were used for computational and visualization tasks: Marvinsketch 24.3.2, Avogadro 1.2.0, ORCA 6.0.1., Discovery Studio Visualizer v24.1.0.23298, Chemcraft 1.8, Yasara 21.12.19, Multiwfn 3.8, VMD 1.9.4a53, Gnuplot 5.4, GIMP 3.0.2, IrfanView 4.70, Ghostscript 10.05.0, Winmostar 11.11.5 with Cgynwin environement, GROMACS 2025.0, Origin 10.2.0.196.

## 5. Conclusions

In this study, theoretical investigations and molecular recognition of a molecularly imprinted polymer (MIP) for the targeted isolation of mitragynine were conducted using computational methods, which were subsequently verified through laboratory experiments. The computational framework employed a host–guest interaction model, considering factors such as binding affinity, intermolecular hydrogen-bond formation, complexation energy, thermodynamic parameters, frontier molecular orbital (FMO) analysis, and monomer compatibility. Based on these criteria, methacrylic acid (MAA) and methanol were identified as the optimal monomer and solvent, respectively, for the complex formation. The non-covalent interactions in the complex were thoroughly investigated using a variety of complementary methods, including QTAIM, NCI-RDG, IRI, IGM, and the IBSIW index. The results indicated that the N5 atom in mitragynine and the H71 atom in methacrylic acid were primarily responsible for the key intermolecular bonds in the host–guest complex formation. Association constant studies confirmed that methanol was the optimal solvent for complexation, exhibiting the highest Ka value. Additionally, the Job’s plot analysis revealed that the predominant complex structure at equilibrium was a 1:3 ratio of template (mitragynine) to functional monomer (methacrylic acid). The radial distribution function analysis from molecular dynamics simulations of the pre-polymerization complex further validated the 1:3 T-FM ratio as optimal for laboratory synthesis. This was corroborated by the host–guest interaction observed in the molecular dynamics study. The combined results from the computational and pre-experimental work demonstrate that computational investigations can provide valuable insights to rationalize and optimize MIP design. These findings offer a foundation for the selective isolation of mitragynine from kratom plants and highlight the utility of computational methods in guiding future experimental synthesis.

## Figures and Tables

**Table 1:** Structure parameterization of mitragynine (Exp. = Experimental, Comp. = Computational, Diff. = Difference).

Distance (Å)	Exp.	Comp.	Diff. (%)	Angle (°)	Experimental	Computational	% Diff.
C27-O1	1.434	1.42	0.71	N5-C7-C12	108.46	108.56	0.09
O1-C21	1.375	1.38	0.34	N5-C7-C10	109.44	109.76	0.30
C21-C25	1.380	1.38	0.23	C12-C7-C10	114.45	112.82	1.42
C25-C26	1.410	1.40	0.51	C25-C21-C18	119.27	119.68	0.34
C26-C24	1.381	1.38	0.28	N6-C19-C24	129.34	130.28	0.73
C24-C19	1.401	1.39	0.64	N6-C19-C18	107.59	107.11	0.45
C19-C18	1.403	1.42	1.09	C14-C12-N6	110.71	109.85	0.78
C21-C18	1.410	1.40	0.69	C14-C12-C7	125.74	125.69	0.04
C18-C14	1.435	1.43	0.09	N6-C12-C7	123.47	124.45	0.79
C14-C12	1.359	1.36	0.34	C7-C10-C9	108.83	110.08	1.15
C12-N6	1.378	1.38	0.11	N5-C13-C15	111.38	111.27	0.10
C19-N6	1.385	1.38	0.58	C22-C17-C9	129.11	126.86	1.75
C14-C15	1.500	1.49	0.45	N5-C11-C8	111.97	111.47	0.44
C15-C13	1.533	1.53	0.29	C11-C8-C16	113.32	112.92	0.35
C13-N5	1.479	1.46	1.31	C11-C8-C9	109.77	107.75	1.84
N5-C7	1.481	1.46	1.14	C9-C8-C16	112.13	114.08	1.73
C12-C7	1.500	1.49	0.57	C12-C14-C18	106.27	106.84	0.54
C7-C10	1.528	1.53	0.22	C12-C14-C15	121.23	121.14	0.07
C10-C9	1.538	1.53	0.33	C18-C14-C15	132.39	132.00	0.30
C9-C8	1.553	1.55	0.18	C24-C26-C25	121.73	121.31	0.35
C8-C11	1.530	1.53	0.12	C17-C23-O3	123.94	122.43	1.22
N5-C11	1.478	1.45	1.62	C19-C18-C21	118.26	118.18	0.07
C8-C16	1.530	1.53	0.23	C19-C18-C14	107.4	107.07	0.30
C16-C20	1.517	1.53	0.71	C21-C18-C14	134.32	134.74	0.31
C9-C17	1.512	1.51	0.18	C21-C25-C26	120.73	120.79	0.05
C17-C22	1.497	1.48	1.17	C14-C15-C13	109.66	108.98	0.62
C22-O4	1.200	1.21	1.11	C24-C19-C18	123.07	122.61	0.38
C22-O2	1.346	1.35	0.33	C26-C4-C19	116.9	117.43	0.46
O2-C28	1.447	1.43	1.01	C8-C16-C20	114.17	113.87	0.26
C17-C23	1.334	1.34	0.82	O2-C22-O4	122.76	121.73	0.84
C23-O3	1.348	1.34	0.42	O4-C22-C17	124.37	125.23	0.69
O3-C29	1.444	1.43	1.16	O2-C22-C17	112.86	113.04	0.16
				C10-C9-C17	117.18	116.48	0.60
				C10-C9-C8	110.24	110.82	0.53
				C13-N5-C7	111.39	112.87	1.33
				C19-N6-C12	108.02	109.13	1.02
				C28-O2-C22	114.73	115.81	0.94

**Table 2:** δg index percentage of mitragynine (a), methacrylic acid (b), and complex 1 (c).

a	Atom	Vacuum	Acetone	Acetonitrile	Chloroform	Dichloromethane	Methanol
	5	7.63	9.41	9.50	9.19	9.41	9.83
	14	6.37	6.52	6.52	6.47	6.51	6.27
	18	5.48	4.96	4.93	4.93	4.90	4.78
	40	5.48	6.50	6.55	6.37	6.51	6.32
	12	5.47	5.39	5.38	5.40	5.39	5.09
	15	5.05	5.81	5.85	5.70	5.81	5.68
	42	4.59	4.52	4.52	4.65	4.58	4.52
	33	4.50	3.91	3.93	4.12	4.00	3.83
	21	4.35	3.85	3.82	3.78	3.76	3.86
	19	3.91	3.22	3.17	3.24	3.17	3.00
b	Atom	Vacuum	Acetone	Acetonitrile	Chloroform	Dichloromethane	Methanol
	71	25.16	28.06	28.25	27.91	28.23	28.65
	68	14.75	28.06	14.60	27.91	14.69	14.24
	70	14.52	15.74	15.82	15.76	15.86	15.90
	69	13.60	11.75	11.67	12.27	11.96	11.28
	62	7.90	6.38	6.36	6.41	6.30	5.97
	64	6.78	6.38	6.53	6.46	6.45	6.54
	60	5.45	4.77	4.75	4.73	4.69	4.59
	65	4.72	4.97	4.93	4.79	4.83	5.33
	67	2.51	2.71	2.68	2.63	2.66	2.91
	63	1.95	1.77	1.75	1.74	1.73	1.73
c	Bond	Vacuum	Acetone	Acetonitrile	Chloroform	Dichloromethane	Methanol
	5–71	4.55	5.59	5.65	5.46	5.59	5.88
	33–69	2.21	1.83	1.82	1.95	1.87	1.74
	13–71	1.88	2.32	2.34	2.26	2.31	1.74
	5–70	1.83	2.30	2.33	2.24	2.30	1.74
	7–71	1.71	1.91	1.94	2.24	1.92	2.00
	40–70	1.48	1.59	1.59	1.59	1.60	1.44
	15–71	1.43	1.61	1.62	1.60	1.62	1.58
	42–71	1.43	1.43	1.43	1.46	1.45	1.38
	14–68	1.42	1.55	1.55	1.54	1.55	1.49
	15–70	1.42	1.56	1.56	1.54	1.56	1.47

## Data Availability

The original contributions presented in this study are included in the article/Supplementary Materials. Further inquiries can be directed to the corresponding author.

## Supplementary Materials

The following supporting information can be downloaded at: https://www.mdpi.com/article/10.3390/molecules31040610/s1, Table S1: Functional monomers used in computational study; Table S2: Intermolecular interactions in mitragynine–monomer complex; Table S3: FMO parameters of all complexes; Table S4: QTAIM analysis of all systems in complex 1; Figure S1: Bond critical point of complex 1; Figure S2: RDG isosurface of complex 1; Figure S3: Binding Mode of Mitragynine with Multi-Monomer MAA; Figure S4: Molecular dynamic packing system; Figure S5: Molecular dynamics analysis.

## Abbreviations

The following abbreviations are used in this manuscript:

MIP	Molecularly Imprinted Polymer
UAE	Ultrasound-Assisted Extraction
MAE	Microwave-Assisted Extraction
PEF	Pulse Electric Field Extraction
EAE	Enzyme-Assisted Extraction
SFE	Supercritical Fluid Extraction
ASE	Accelerated Solvent Extraction
FM	Functional Monomer
QTAIM	Quantum theory of atoms in molecules
NCI-RDG	Non-covalent interactions-reduced density gradient
IRI	Interaction region indicator
IGM	Independent gradient model
AM1-BCC	Austin Model 1–Bond Charge Correction
GAFF-2	General Amber Force Field 2
SMD	Solvent Model Density
LUMO	Lowest Unoccupied Molecular Orbital
HOMO	Highest Occupied Molecular Orbital
BCP	Bond Critical Point
RGB	Red-Green-Blue
Ka	Association Constant
MD	Molecular Dynamics
EM	Energy Minimization
RMSD	Root Mean Square Deviation
RMSF	Root Mean Square Fluctuation
RDF	Radial Distribution Function
MAA	Methacrylic Acid
EGDMA	Ethylene Glycol Dimethacrylate
