# Supplementary material for: Unravelling Rational Design of Molecularly Imprinted Polymer for Selective Mitragynine Isolation from Kratom: Quantum Mechanical, Molecular Dynamics, and Experimental Insights
Source: Molecules. 2026 Feb 10;31(4):610. doi: 10.3390/molecules31040610 (PMC12943084; doi:10.3390/molecules31040610)
Supplement: Supplementary file 1 [file molecules-31-00610-s001.zip › molecules-4124046-supplementary.pdf]

Supplementary Materials

Unravelling Rational Design Molecularly Imprinted Polymer for Selective Mitragynine Isolation from Kratom: Quantum Mechanical, Molecular Dynamics, and Experimental Insights

Table S1. Functional monomers used in computational study

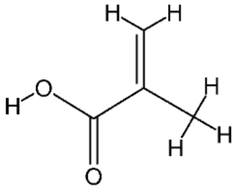 Methacrylic acid (FM-1)	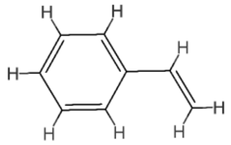 Styrene (FM-13)	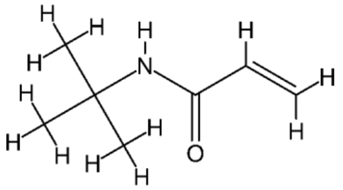 N-(t-butyl)acrilamide (FM-25)
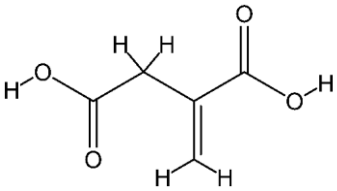 Itaconic acid (FM-2)	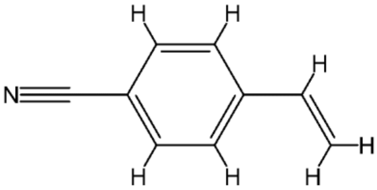 4-Cyanostyrene (FM-14)	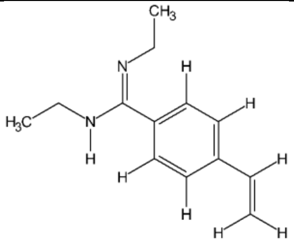 N,N′-Diethyl-4-vinylbenzamidine (FM-26)
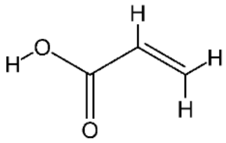 Acrylic acid (FM-3)	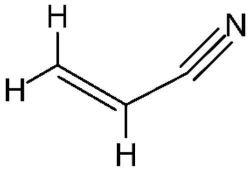 Acrylonitrile (FM-15)	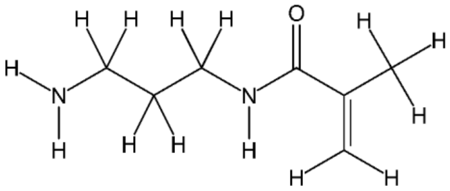 N-(3-aminopropyl) methacrylamide (FM-27)
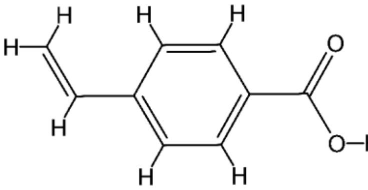 p-vinylbenzoic acid (FM-4)	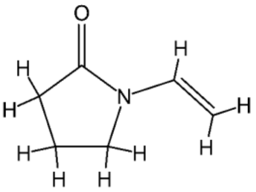 N-vinyl-2-pyrrolidinone (FM-16)	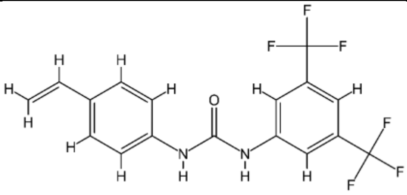 N-(3,5-Bis(trifluoromethyl)phenyl)- N′-(4-vinylphenyl)urea (FM-28)
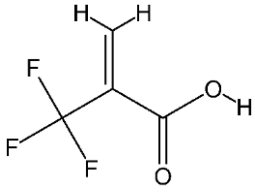 2-(Trifluoromethyl)-acrylic acid (FM-5)	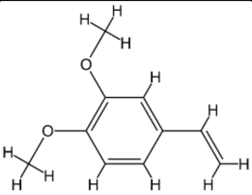 4-Vinylveratrole (FM-17)	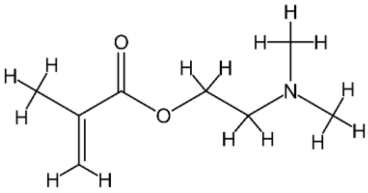 N,N-diethylaminomethylmethacrylate (FM-29)
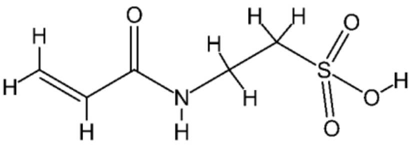 2-Acrylamido-1-ethanesulfonic acid (FM-6)	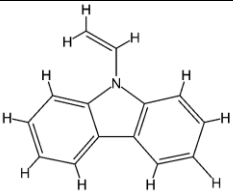 9-Vinylcarbazol (FM-18)	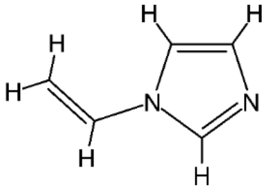 1-Vinylimidazole (FM-30)
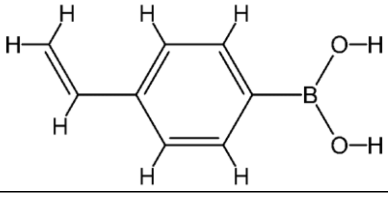	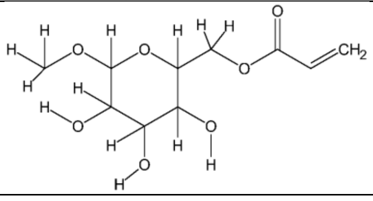	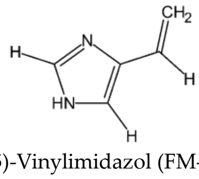 4(5)-Vinylimidazol (FM-31)

4-vinylbenzeneboronic acid (FM-7)	Methyl-6-O-acrylate-α-D glucopyranoside (FM-19)	
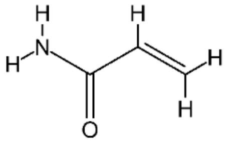 Acrylamide (FM-8)	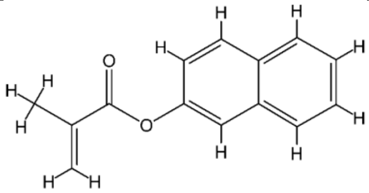 2-Naphthylmethacrylate (FM-20)	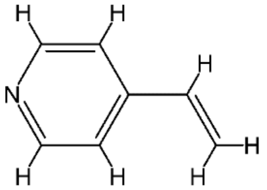 4-Vinylpyridine (FM-32)
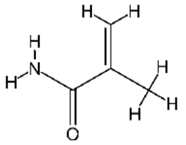 Methacrylamide (FM9)	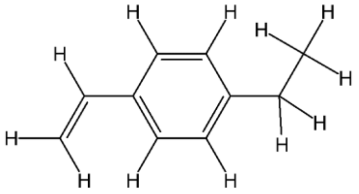 4-ethylstyrene (FM-21)	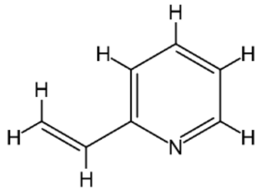 2-Vinylpyridin (FM-33)
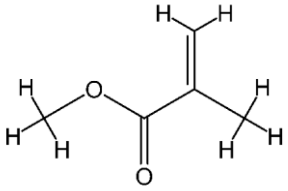 Methyl methacrylate (FM-10)	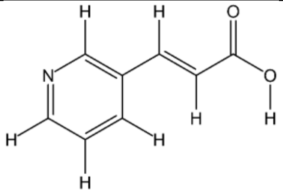 trans-3-(3-pirydyl)-acrylic acid (FM-22)	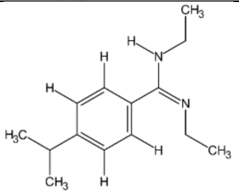 N,N′-diethyl-4-styrylamidine (FM-34)
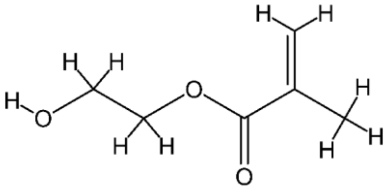 2-Hydroxyethylmethacrylate (FM-11)	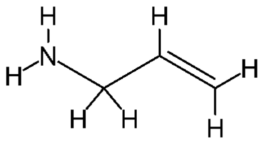 Allylamine (FM-23)	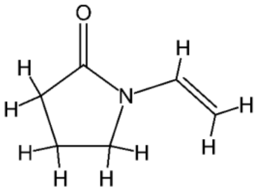 N-vinylpyrrolidone (FM-35)
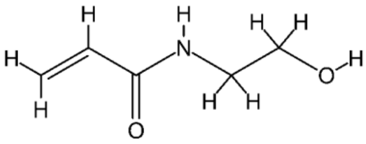 N-(2-hydroxyethyl)acrylamide (FM-12)	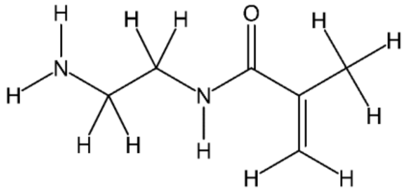 N-(2-aminoethyl)methacrylamide (FM-24)	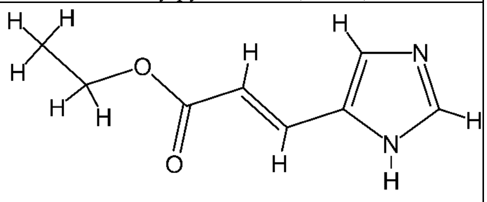 Urocanic etyl ester (FM-36)

Table S2. Intermolecular interactions in mitragynine-monomer complex

Complex	Interaction type			
	**Hydrogen Bond**	**Hydrophobic**	**Halogen Bond**	**Other**
1	+	+	-	-
2	+	-	-	-
3	+	+	-	-
4	-	+	-	-
5	+	+	+	-
6	+	-	-	-
7	-	+	-	-
8	+	+	-	-
9	+	+	-	-
10	+	-	-	-
11	+	+	-	-
12	+	+	-	-
13	-	+	-	-
14	-	+	-	-
15	-	+	-	-

16	-	+	-	-
17	-	+	-	-
18	-	-	-	-
19	+	-	-	-
20	+	+	-	-
21	-	+	-	-
22	-	+	-	-
23	-	+	-	-
24	+	+	-	-
25	+	+	-	-
26	-	+	-	-
27	-	+	-	-
28	+	-	+	-
29	+	+	-	-
30	-	+	-	-
31	+	+	-	-
32	+	+	-	-
33	+	+	-	-
34	-	+	-	-
35	-	+	-	-
36	-	+	-	-

Table S3. FMO parameters of all complexes

Complex	Condition	Eg	EA	IP	η	S	μ	ω	SE	X
1	vacuum	4.140	0.910	5.050	2.070	0.242	-2.980	9.192	-9.192	2.980
	acetone	4.063	1.020	5.083	2.031	0.246	-3.052	9.459	-9.459	3.052
	acetonitrile	4.054	1.038	5.092	2.027	0.247	-3.065	9.523	-9.523	3.065
	chloroform	4.064	1.007	5.070	2.032	0.246	-3.039	9.380	-9.380	3.039
	dichlorometane	4.059	1.021	5.080	2.029	0.246	-3.050	9.442	-9.442	3.050
	methanol	3.969	1.207	5.176	1.984	0.252	-3.192	10.108	-10.108	3.192
2	vacuum	3.989	1.406	5.395	1.994	0.251	-3.400	11.530	-11.530	3.400
	acetone	3.946	1.347	5.293	1.973	0.253	-3.320	10.877	-10.877	3.320
	acetonitrile	3.944	1.352	5.296	1.972	0.254	-3.324	10.892	-10.892	3.324
	chloroform	3.945	1.366	5.311	1.972	0.254	-3.339	10.992	-10.992	3.339
	dichlorometane	3.942	1.358	5.301	1.971	0.254	-3.330	10.927	-10.927	3.330
	methanol	3.844	1.505	5.348	1.922	0.260	-3.426	11.281	-11.281	3.426
3	vacuum	3.821	1.409	5.230	1.910	0.262	-3.320	10.525	-10.525	3.320
	acetone	3.849	1.441	5.290	1.925	0.260	-3.366	10.900	-10.900	3.366
	acetonitrile	3.849	1.448	5.297	1.925	0.260	-3.372	10.944	-10.944	3.372
	chloroform	3.829	1.440	5.269	1.914	0.261	-3.354	10.770	-10.770	3.354
	dichlorometane	3.843	1.442	5.284	1.921	0.260	-3.363	10.866	-10.866	3.363
	methanol	3.820	1.515	5.335	1.910	0.262	-3.425	11.203	-11.203	3.425
4	vacuum	3.552	1.726	5.277	1.776	0.282	-3.502	10.887	-10.887	3.502
	acetone	3.517	1.772	5.289	1.758	0.284	-3.530	10.957	-10.957	3.530
	acetonitrile	3.519	1.775	5.294	1.759	0.284	-3.534	10.989	-10.989	3.534
	chloroform	3.499	1.779	5.278	1.749	0.286	-3.528	10.889	-10.889	3.528
	dichlorometane	3.509	1.776	5.285	1.755	0.285	-3.530	10.935	-10.935	3.530
	methanol	3.484	1.846	5.330	1.742	0.287	-3.588	11.214	-11.214	3.588
5	vacuum	3.357	2.029	5.386	1.678	0.298	-3.708	11.536	-11.536	3.708

	acetone	3.453	1.852	5.305	1.726	0.290	-3.578	11.052	-11.052	3.578
	acetonitrile	3.460	1.846	5.306	1.730	0.289	-3.576	11.063	-11.063	3.576
	chloroform	3.442	1.879	5.321	1.721	0.291	-3.600	11.150	-11.150	3.600
	dichlorometane	3.448	1.863	5.311	1.724	0.290	-3.587	11.090	-11.090	3.587
	methanol	3.432	1.910	5.342	1.716	0.291	-3.626	11.282	-11.282	3.626
6	vacuum	4.146	0.958	5.103	2.073	0.241	-3.030	9.518	-9.518	3.030
	acetone	4.037	1.055	5.092	2.019	0.248	-3.074	9.536	-9.536	3.074
	acetonitrile	4.032	1.064	5.096	2.016	0.248	-3.080	9.562	-9.562	3.080
	chloroform	4.054	1.036	5.090	2.027	0.247	-3.063	9.507	-9.507	3.063
	dichlorometane	4.032	1.052	5.084	2.016	0.248	-3.068	9.491	-9.491	3.068
	methanol	3.948	1.199	5.147	1.974	0.253	-3.173	9.939	-9.939	3.173
7	vacuum	3.970	1.337	5.307	1.985	0.252	-3.322	10.954	-10.954	3.322
	acetone	3.901	1.385	5.285	1.950	0.256	-3.335	10.846	-10.846	3.335
	acetonitrile	3.908	1.383	5.291	1.954	0.256	-3.337	10.881	-10.881	3.337
	chloroform	3.892	1.371	5.263	1.946	0.257	-3.317	10.707	-10.707	3.317
	dichlorometane	3.895	1.381	5.276	1.948	0.257	-3.329	10.790	-10.790	3.329
	methanol	3.933	1.395	5.328	1.967	0.254	-3.361	11.109	-11.109	3.361
8	vacuum	4.458	0.916	5.373	2.229	0.224	-3.144	11.018	-11.018	3.144
	acetone	4.130	1.055	5.185	2.065	0.242	-3.120	10.050	-10.050	3.120
	acetonitrile	4.114	1.069	5.183	2.057	0.243	-3.126	10.050	-10.050	3.126
	chloroform	4.174	1.049	5.223	2.087	0.240	-3.136	10.262	-10.262	3.136
	dichlorometane	4.143	1.057	5.200	2.071	0.241	-3.129	10.139	-10.139	3.129
	methanol	4.009	1.217	5.226	2.004	0.249	-3.221	10.400	-10.400	3.221
9	vacuum	4.179	1.037	5.216	2.090	0.239	-3.126	10.213	-10.213	3.126
	acetone	4.369	0.917	5.286	2.185	0.229	-3.102	10.507	-10.507	3.102
	acetonitrile	4.369	0.927	5.297	2.185	0.229	-3.112	10.579	-10.579	3.112
	chloroform	4.338	0.921	5.259	2.169	0.231	-3.090	10.358	-10.358	3.090
	dichlorometane	4.347	0.934	5.280	2.173	0.230	-3.107	10.491	-10.491	3.107
	methanol	4.243	1.082	5.324	2.121	0.236	-3.203	10.883	-10.883	3.203
10	vacuum	4.010	0.964	4.974	2.005	0.249	-2.969	8.839	-8.839	2.969
	acetone	4.035	1.030	5.066	2.018	0.248	-3.048	9.373	-9.373	3.048
	acetonitrile	4.028	1.047	5.075	2.014	0.248	-3.061	9.433	-9.433	3.061
	chloroform	4.010	1.035	5.045	2.005	0.249	-3.040	9.263	-9.263	3.040
	dichlorometane	4.018	1.042	5.060	2.009	0.249	-3.051	9.350	-9.350	3.051
	methanol	3.870	1.265	5.135	1.935	0.258	-3.200	9.908	-9.908	3.200
11	vacuum	3.984	1.168	5.152	1.992	0.251	-3.160	9.945	-9.945	3.160
	acetone	4.128	1.139	5.267	2.064	0.242	-3.203	10.586	-10.586	3.203
	acetonitrile	4.117	1.156	5.272	2.058	0.243	-3.214	10.631	-10.631	3.214
	chloroform	4.062	1.171	5.233	2.031	0.246	-3.202	10.412	-10.412	3.202
	dichlorometane	4.086	1.166	5.252	2.043	0.245	-3.209	10.522	-10.522	3.209
	methanol	3.997	1.319	5.316	1.998	0.250	-3.317	10.994	-10.994	3.317
12	vacuum	4.193	0.923	5.116	2.097	0.238	-3.020	9.558	-9.558	3.020
	acetone	4.108	0.997	5.105	2.054	0.243	-3.051	9.558	-9.558	3.051
	acetonitrile	4.097	1.009	5.106	2.048	0.244	-3.057	9.575	-9.575	3.057
	chloroform	4.114	0.986	5.100	2.057	0.243	-3.043	9.522	-9.522	3.043
	dichlorometane	4.105	0.997	5.102	2.052	0.244	-3.049	9.541	-9.541	3.049

	methanol	3.961	1.147	5.107	1.980	0.252	-3.127	9.682	-9.682	3.127
13	vacuum	4.137	1.031	5.168	2.069	0.242	-3.099	9.936	-9.936	3.099
	acetone	4.204	1.071	5.275	2.102	0.238	-3.173	10.581	-10.581	3.173
	acetonitrile	4.207	1.075	5.283	2.104	0.238	-3.179	10.629	-10.629	3.179
	chloroform	4.196	1.045	5.241	2.098	0.238	-3.143	10.362	-10.362	3.143
	dichlorometane	4.198	1.063	5.261	2.099	0.238	-3.162	10.496	-10.496	3.162
	methanol	4.238	1.078	5.316	2.119	0.236	-3.197	10.829	-10.829	3.197
14	vacuum	3.570	1.813	5.383	1.785	0.280	-3.598	11.556	-11.556	3.598
	acetone	3.508	1.789	5.297	1.754	0.285	-3.543	11.011	-11.011	3.543
	acetonitrile	3.512	1.787	5.299	1.756	0.285	-3.543	11.019	-11.019	3.543
	chloroform	3.493	1.813	5.305	1.746	0.286	-3.559	11.059	-11.059	3.559
	dichlorometane	3.499	1.802	5.301	1.750	0.286	-3.551	11.032	-11.032	3.551
	methanol	3.547	1.787	5.335	1.774	0.282	-3.561	11.245	-11.245	3.561
15	vacuum	3.709	1.442	5.151	1.854	0.270	-3.297	10.076	-10.076	3.297
	acetone	3.781	1.337	5.118	1.890	0.264	-3.228	9.846	-9.846	3.228
	acetonitrile	3.779	1.342	5.121	1.890	0.265	-3.232	9.867	-9.867	3.232
	chloroform	3.767	1.355	5.122	1.884	0.265	-3.238	9.877	-9.877	3.238
	dichlorometane	3.771	1.350	5.121	1.885	0.265	-3.235	9.867	-9.867	3.235
	methanol	3.817	1.345	5.162	1.908	0.262	-3.253	10.100	-10.100	3.253
16	vacuum	4.323	0.719	5.042	2.161	0.231	-2.881	8.968	-8.968	2.881
	acetone	4.315	0.763	5.078	2.157	0.232	-2.921	9.203	-9.203	2.921
	acetonitrile	4.307	0.771	5.078	2.154	0.232	-2.924	9.209	-9.209	2.924
	chloroform	4.293	0.754	5.047	2.146	0.233	-2.900	9.027	-9.027	2.900
	dichlorometane	4.311	0.749	5.060	2.156	0.232	-2.905	9.093	-9.093	2.905
	methanol	4.185	0.935	5.120	2.093	0.239	-3.027	9.590	-9.590	3.027
17	vacuum	4.071	1.073	5.144	2.036	0.246	-3.109	9.836	-9.836	3.109
	acetone	4.451	0.803	5.254	2.226	0.225	-3.028	10.206	-10.206	3.028
	acetonitrile	4.453	0.809	5.262	2.227	0.225	-3.036	10.261	-10.261	3.036
	chloroform	4.360	0.867	5.227	2.180	0.229	-3.047	10.120	-10.120	3.047
	dichlorometane	4.422	0.822	5.244	2.211	0.226	-3.033	10.171	-10.171	3.033
	methanol	4.324	0.966	5.290	2.162	0.231	-3.128	10.575	-10.575	3.128
18	vacuum	3.998	1.035	5.034	1.999	0.250	-3.035	9.204	-9.204	3.035
	acetone	4.166	1.052	5.218	2.083	0.240	-3.135	10.238	-10.238	3.135
	acetonitrile	4.171	1.056	5.227	2.085	0.240	-3.141	10.290	-10.290	3.141
	chloroform	4.146	1.033	5.179	2.073	0.241	-3.106	10.002	-10.002	3.106
	dichlorometane	4.163	1.045	5.208	2.081	0.240	-3.126	10.172	-10.172	3.126
	methanol	4.194	1.045	5.240	2.097	0.238	-3.143	10.356	-10.356	3.143
19	vacuum	3.598	1.588	5.186	1.799	0.278	-3.387	10.317	-10.317	3.387
	acetone	3.791	1.450	5.240	1.895	0.264	-3.345	10.604	-10.604	3.345
	acetonitrile	3.800	1.450	5.250	1.900	0.263	-3.350	10.661	-10.661	3.350
	chloroform	3.747	1.485	5.232	1.874	0.267	-3.358	10.564	-10.564	3.358
	dichlorometane	3.775	1.464	5.239	1.888	0.265	-3.352	10.603	-10.603	3.352
	methanol	3.785	1.530	5.315	1.892	0.264	-3.423	11.084	-11.084	3.423
20	vacuum	3.718	1.484	5.203	1.859	0.269	-3.343	10.391	-10.391	3.343
	acetone	3.857	1.393	5.250	1.929	0.259	-3.322	10.639	-10.639	3.322
	acetonitrile	3.864	1.394	5.258	1.932	0.259	-3.326	10.687	-10.687	3.326

	chloroform	3.817	1.421	5.238	1.908	0.262	-3.329	10.576	-10.576	3.329
	dichlorometane	3.842	1.407	5.249	1.921	0.260	-3.328	10.637	-10.637	3.328
	methanol	3.806	1.497	5.304	1.903	0.263	-3.400	11.003	-11.003	3.400
21	vacuum	4.162	1.016	5.177	2.081	0.240	-3.096	9.975	-9.975	3.096
	acetone	4.278	0.995	5.273	2.139	0.234	-3.134	10.503	-10.503	3.134
	acetonitrile	4.280	1.000	5.280	2.140	0.234	-3.140	10.551	-10.551	3.140
	chloroform	4.271	0.969	5.241	2.136	0.234	-3.105	10.295	-10.295	3.105
	dichlorometane	4.277	0.984	5.260	2.138	0.234	-3.122	10.421	-10.421	3.122
	methanol	4.313	1.002	5.314	2.156	0.232	-3.158	10.753	-10.753	3.158
22	vacuum	3.110	2.242	5.353	1.555	0.322	-3.797	11.213	-11.213	3.797
	acetone	3.236	2.033	5.269	1.618	0.309	-3.651	10.785	-10.785	3.651
	acetonitrile	3.244	2.029	5.273	1.622	0.308	-3.651	10.811	-10.811	3.651
	chloroform	3.203	2.090	5.293	1.602	0.312	-3.691	10.912	-10.912	3.691
	dichlorometane	3.213	2.075	5.288	1.607	0.311	-3.682	10.889	-10.889	3.682
	methanol	3.217	2.117	5.334	1.608	0.311	-3.725	11.161	-11.161	3.725
23	vacuum	4.391	0.898	5.288	2.195	0.228	-3.093	10.501	-10.501	3.093
	acetone	4.553	0.766	5.319	2.276	0.220	-3.042	10.535	-10.535	3.042
	acetonitrile	4.550	0.773	5.323	2.275	0.220	-3.048	10.570	-10.570	3.048
	chloroform	4.492	0.814	5.306	2.246	0.223	-3.060	10.513	-10.513	3.060
	dichlorometane	4.524	0.791	5.315	2.262	0.221	-3.053	10.540	-10.540	3.053
	methanol	4.400	0.958	5.358	2.200	0.227	-3.158	10.970	-10.970	3.158
24	vacuum	3.933	1.296	5.229	1.966	0.254	-3.263	10.466	-10.466	3.263
	acetone	4.508	0.801	5.309	2.254	0.222	-3.055	10.516	-10.516	3.055
	acetonitrile	4.508	0.800	5.308	2.254	0.222	-3.054	10.514	-10.514	3.054
	chloroform	4.385	0.927	5.313	2.193	0.228	-3.120	10.671	-10.671	3.120
	dichlorometane	4.459	0.854	5.314	2.230	0.224	-3.084	10.603	-10.603	3.084
	methanol	4.347	0.985	5.332	2.174	0.230	-3.158	10.839	-10.839	3.158
25	vacuum	4.439	1.069	5.508	2.220	0.225	-3.289	12.004	-12.004	3.289
	acetone	4.386	0.928	5.314	2.193	0.228	-3.121	10.678	-10.678	3.121
	acetonitrile	4.365	0.943	5.308	2.183	0.229	-3.125	10.658	-10.658	3.125
	chloroform	4.411	0.949	5.360	2.206	0.227	-3.155	10.974	-10.974	3.155
	dichlorometane	4.394	0.942	5.335	2.197	0.228	-3.138	10.820	-10.820	3.138
	methanol	4.255	1.092	5.348	2.128	0.235	-3.220	11.030	-11.030	3.220
26	vacuum	4.068	1.300	5.368	2.034	0.246	-3.334	11.305	-11.305	3.334
	acetone	4.033	1.253	5.287	2.017	0.248	-3.270	10.782	-10.782	3.270
	acetonitrile	4.036	1.253	5.289	2.018	0.248	-3.271	10.797	-10.797	3.271
	chloroform	4.009	1.289	5.298	2.005	0.249	-3.294	10.874	-10.874	3.294
	dichlorometane	4.026	1.265	5.290	2.013	0.248	-3.277	10.810	-10.810	3.277
	methanol	4.145	1.179	5.323	2.072	0.241	-3.251	10.952	-10.952	3.251
27	vacuum	3.793	1.243	5.036	1.897	0.264	-3.140	9.348	-9.348	3.140
	acetone	4.343	0.876	5.219	2.172	0.230	-3.048	10.085	-10.085	3.048
	acetonitrile	4.339	0.882	5.222	2.170	0.230	-3.052	10.106	-10.106	3.052
	chloroform	4.230	0.944	5.174	2.115	0.236	-3.059	9.896	-9.896	3.059
	dichlorometane	4.316	0.884	5.200	2.158	0.232	-3.042	9.987	-9.987	3.042
	methanol	4.267	0.971	5.238	2.134	0.234	-3.104	10.281	-10.281	3.104
28	vacuum	3.368	1.537	4.904	1.684	0.297	-3.220	8.732	-8.732	3.220

	acetone	3.818	1.242	5.060	1.909	0.262	-3.151	9.479	-9.479	3.151
	acetonitrile	3.833	1.237	5.070	1.917	0.261	-3.154	9.530	-9.530	3.154
	chloroform	3.720	1.293	5.013	1.860	0.269	-3.153	9.248	-9.248	3.153
	dichlorometane	3.781	1.261	5.041	1.890	0.265	-3.151	9.385	-9.385	3.151
	methanol	3.860	1.254	5.114	1.930	0.259	-3.184	9.782	-9.782	3.184
29	vacuum	3.871	1.149	5.021	1.936	0.258	-3.085	9.212	-9.212	3.085
	acetone	4.123	1.073	5.196	2.061	0.243	-3.135	10.126	-10.126	3.135
	acetonitrile	4.117	1.087	5.204	2.058	0.243	-3.146	10.184	-10.184	3.146
	chloroform	4.037	1.117	5.155	2.019	0.248	-3.136	9.926	-9.926	3.136
	dichlorometane	4.087	1.094	5.181	2.044	0.245	-3.137	10.058	-10.058	3.137
	methanol	3.935	1.308	5.243	1.968	0.254	-3.276	10.557	-10.557	3.276
30	vacuum	4.173	1.085	5.258	2.086	0.240	-3.171	10.491	-10.491	3.171
	acetone	4.501	0.783	5.283	2.250	0.222	-3.033	10.351	-10.351	3.033
	acetonitrile	4.511	0.779	5.290	2.255	0.222	-3.034	10.382	-10.382	3.034
	chloroform	4.405	0.866	5.271	2.202	0.227	-3.068	10.367	-10.367	3.068
	dichlorometane	4.462	0.817	5.279	2.231	0.224	-3.048	10.366	-10.366	3.048
	methanol	4.364	0.956	5.320	2.182	0.229	-3.138	10.740	-10.740	3.138
31	vacuum	4.557	0.776	5.333	2.279	0.219	-3.055	10.630	-10.630	3.055
	acetone	4.520	0.749	5.269	2.260	0.221	-3.009	10.233	-10.233	3.009
	acetonitrile	4.512	0.765	5.278	2.256	0.222	-3.021	10.299	-10.299	3.021
	chloroform	4.484	0.776	5.260	2.242	0.223	-3.018	10.210	-10.210	3.018
	dichlorometane	4.511	0.756	5.267	2.255	0.222	-3.012	10.229	-10.229	3.012
	methanol	4.355	0.951	5.306	2.177	0.230	-3.128	10.655	-10.655	3.128
32	vacuum	3.860	1.391	5.251	1.930	0.259	-3.321	10.643	-10.643	3.321
	acetone	3.806	1.476	5.282	1.903	0.263	-3.379	10.866	-10.866	3.379
	acetonitrile	3.810	1.478	5.288	1.905	0.262	-3.383	10.900	-10.900	3.383
	chloroform	3.805	1.457	5.262	1.902	0.263	-3.359	10.735	-10.735	3.359
	dichlorometane	3.802	1.474	5.275	1.901	0.263	-3.374	10.822	-10.822	3.374
	methanol	3.837	1.484	5.322	1.919	0.261	-3.403	11.108	-11.108	3.403
33	vacuum	3.984	1.246	5.230	1.992	0.251	-3.238	10.441	-10.441	3.238
	acetone	4.003	1.282	5.285	2.001	0.250	-3.283	10.788	-10.788	3.283
	acetonitrile	4.005	1.287	5.292	2.002	0.250	-3.290	10.835	-10.835	3.290
	chloroform	3.978	1.281	5.259	1.989	0.251	-3.270	10.635	-10.635	3.270
	dichlorometane	3.992	1.283	5.275	1.996	0.251	-3.279	10.732	-10.732	3.279
	methanol	4.014	1.311	5.326	2.007	0.249	-3.318	11.051	-11.051	3.318
34	vacuum	4.474	0.961	5.435	2.237	0.223	-3.198	11.440	-11.440	3.198
	acetone	4.528	0.770	5.298	2.264	0.221	-3.034	10.419	-10.419	3.034
	acetonitrile	4.533	0.773	5.306	2.267	0.221	-3.039	10.467	-10.467	3.039
	chloroform	4.499	0.824	5.324	2.250	0.222	-3.074	10.629	-10.629	3.074
	dichlorometane	4.518	0.795	5.313	2.259	0.221	-3.054	10.536	-10.536	3.054
	methanol	4.379	0.953	5.332	2.190	0.228	-3.143	10.812	-10.812	3.143
35	vacuum	4.322	0.719	5.042	2.161	0.231	-2.881	8.967	-8.967	2.881
	acetone	4.313	0.763	5.076	2.157	0.232	-2.920	9.192	-9.192	2.920
	acetonitrile	4.307	0.770	5.077	2.154	0.232	-2.923	9.202	-9.202	2.923
	chloroform	4.293	0.754	5.046	2.146	0.233	-2.900	9.026	-9.026	2.900
	dichlorometane	4.312	0.748	5.060	2.156	0.232	-2.904	9.091	-9.091	2.904

	methanol	4.186	0.934	5.120	2.093	0.239	-3.027	9.590	-9.590	3.027
36	vacuum	3.568	1.743	5.312	1.784	0.280	-3.528	11.101	-11.101	3.528
	acetone	3.722	1.584	5.306	1.861	0.269	-3.445	11.043	-11.043	3.445
	acetonitrile	3.715	1.592	5.307	1.857	0.269	-3.450	11.052	-11.052	3.450
	chloroform	3.673	1.647	5.320	1.836	0.272	-3.483	11.142	-11.142	3.483
	dichlorometane	3.701	1.617	5.318	1.851	0.270	-3.467	11.123	-11.123	3.467
	methanol	3.619	1.730	5.350	1.810	0.276	-3.540	11.338	11.338	3.540

Table S4. QTAIM analysis of all systems in complex 1

System	Interaction	BCP	Condition	*P*	GBCP	VBCP	HBCP	(∇²p)	λ1	λ2	λ3	∣V/G∣
1	5(N) - 71(H)	93	Vacuum	0.0412	0.0254	-0.0323	-0.0068	0.0744	-0.0525	0.0161	0.1108	1.2692
		93	Acetone	0.0560	0.0333	-0.0488	-0.0155	0.0710	-0.0792	-0.0087	0.1589	1.4666
		94	Acetonitrile	0.0575	0.0340	-0.0505	-0.0165	0.0701	-0.0827	-0.0089	0.1617	1.4850
		94	Chloroform	0.0539	0.0322	-0.0465	-0.0142	0.0722	-0.0765	-0.0041	-0.0041	1.4406
		94	Dichloromethane	0.0559	0.0332	-0.0487	-0.0154	0.0711	-0.0798	-0.0070	0.1580	1.4647
		94	Methanol	0.0703	0.0395	-0.0649	-0.0254	0.0564	-0.1170	-0.0170	-0.0170	1.6427
2	40(H) - 70(O)	94	Vacuum	0.0095	0.0078	-0.0060	0.0018	0.0383	0.0192	0.0105	0.0086	0.7725
		96	Acetone	0.0100	0.0082	-0.0064	0.0018	0.0401	0.0222	0.0065	0.0114	0.7796
		96	Acetonitrile	0.0101	0.0083	-0.0065	0.0018	0.0403	0.0226	0.0064	0.0113	0.7802
		96	Chloroform	0.0100	0.0082	-0.0064	0.0018	0.0401	0.0225	0.0070	0.0107	0.7800
		96	Dichloromethane	0.0101	0.0083	-0.0065	0.0018	0.0403	0.0227	0.0066	0.0111	0.7804
		96	Methanol	0.0098	0.0080	-0.0062	0.0018	0.0392	0.0237	0.0054	0.0101	0.7778
3	71(H) - 42(H)	100	Vacuum	0.0062	0.0044	-0.0033	0.0011	0.0221	0.0277	-0.0030	-0.0026	0.7502
		NA	Acetone	NA	NA	NA	NA	NA	NA	NA	NA	NA
		NA	Acetonitrile	NA	NA	NA	NA	NA	NA	NA	NA	NA
		NA	Chloroform	NA	NA	NA	NA	NA	NA	NA	NA	NA
		NA	Dichloromethane	NA	NA	NA	NA	NA	NA	NA	NA	NA
		NA	Methanol	NA	NA	NA	NA	NA	NA	NA	NA	NA
4	40(H) - 62(H)	105	Vacuum	0.0035	0.0024	-0.0018	0.0006	0.0119	-0.0004	0.0062	0.0061	0.7606
		97	Acetone	0.0045	0.0029	-0.0023	0.0006	0.0137	-0.0015	0.0031	0.0122	0.8015
		97	Acetonitrile	0.0046	0.0029	-0.0023	0.0006	0.0140	-0.0017	0.0030	0.0127	0.8037
		98	Chloroform	0.0043	0.0028	-0.0022	0.0006	0.0133	-0.0014	0.0034	0.0113	0.7965
		97	Dichloromethane	0.0044	0.0028	-0.0023	0.0006	0.0136	-0.0015	0.0030	0.0121	0.8003
		97	Methanol	0.0049	0.0031	-0.0025	0.0006	0.0148	-0.0025	0.0025	0.0148	0.8091
5	70(O) - 14(C)	113	Vacuum	0.0083	0.0067	-0.0051	0.0016	0.0331	0.0057	-0.0024	0.0299	0.7582
		NA	Acetone	NA	NA	NA	NA	NA	NA	NA	NA	NA
		NA	Acetonitrile	NA	NA	NA	NA	NA	NA	NA	NA	NA
		NA	Chloroform	NA	NA	NA	NA	NA	NA	NA	NA	NA
		NA	Dichloromethane	NA	NA	NA	NA	NA	NA	NA	NA	NA
		NA	Methanol	NA	NA	NA	NA	NA	NA	NA	NA	NA

6	70(O) - 42(H)	NA	Vacuum	NA	NA	NA	NA	NA	NA	NA	NA	NA
		NA	Acetone	NA	NA	NA	NA	NA	NA	NA	NA	NA
		NA	Acetonitrile	NA	NA	NA	NA	NA	NA	NA	NA	NA
		NA	Chloroform	NA	NA	NA	NA	NA	NA	NA	NA	NA
		NA	Dichloromethane	NA	NA	NA	NA	NA	NA	NA	NA	NA
		103	Methanol	0.0065	0.0049	-0.0036	0.0013	0.2508	0.0311	-0.0035	-0.0025	0.7292
7	62(H) - 1(O)	127	Vacuum	0.0059	0.0042	-0.0031	0.0011	0.0211	0.0086	-0.0040	0.0166	0.7411
		115	Acetone	0.0037	0.0026	-0.0018	0.0008	0.0134	0.0045	-0.0026	0.0115	0.7096
		114	Acetonitrile	0.0037	0.0026	-0.0018	0.0007	0.0133	0.0045	-0.0026	0.0114	0.7089
		115	Chloroform	0.0039	0.0027	-0.0019	0.0008	0.0137	0.0049	-0.0028	0.0116	0.7139
		113	Dichloromethane	0.0036	0.0025	-0.0018	0.0007	0.0130	0.0045	-0.0025	0.0111	0.7084
		114	Methanol	0.0037	0.0025	-0.0018	0.0007	0.0131	0.0048	-0.0023	0.0106	0.7093
8	42(H) - 69(O)	128	Vacuum	0.0056	0.0037	-0.0028	0.0009	0.0182	0.0119	0.0098	-0.0035	0.7673
		124	Acetone	0.0050	0.0032	-0.0025	0.0007	0.0158	0.0121	0.0067	-0.0030	0.7696
		123	Acetonitrile	0.0049	0.0032	-0.0024	0.0007	0.0155	0.0118	0.0066	-0.0028	0.7695
		122	Chloroform	0.0051	0.0033	-0.0026	0.0008	0.0164	0.0122	0.0073	-0.0031	0.7705
		123	Dichloromethane	0.0049	0.0032	-0.0025	0.0007	0.0158	0.0119	0.0068	-0.0029	0.7693
		127	Methanol	0.0051	0.0033	-0.0026	0.0007	0.0162	0.0120	0.0073	-0.0031	0.7797
9	12(C) - 69(O)	147	Vacuum	0.0065	0.0050	-0.0038	0.0013	0.0252	0.0016	0.0048	0.0188	0.7464
		NA	Acetone	NA	NA	NA	NA	NA	NA	NA	NA	NA
		NA	Acetonitrile	NA	NA	NA	NA	NA	NA	NA	NA	NA
		NA	Chloroform	NA	NA	NA	NA	NA	NA	NA	NA	NA
		NA	Dichloromethane	NA	NA	NA	NA	NA	NA	NA	NA	NA
		NA	Methanol	NA	NA	NA	NA	NA	NA	NA	NA	NA
10	33(H) - 69(O)	150	Vacuum	0.0133	0.0102	-0.0080	0.0022	0.0495	0.0097	-0.0001	0.0399	0.7827
		144	Acetone	0.0100	0.0072	-0.0055	0.0017	0.0355	0.0113	-0.0025	0.0266	0.7688
		143	Acetonitrile	0.0100	0.0072	-0.0055	0.0017	0.0354	0.0114	-0.0024	0.0263	0.7696
		142	Chloroform	0.0109	0.0080	-0.0062	0.0018	0.0392	0.0116	0.0116	0.0300	0.7732
		144	Dichloromethane	0.0103	0.0075	-0.0057	0.0017	0.0366	0.0116	-0.0024	0.0274	0.7707
		146	Methanol	0.0104	0.0074	-0.0058	0.0016	0.0360	0.0098	-0.0023	0.0284	0.7859
11	64(C) - 18(C)	NA	Vacuum	NA	NA	NA	NA	NA	NA	NA	NA	NA
		152	Acetone	0.0041	0.0024	-0.0016	0.0007	0.0123	0.0014	-0.0006	0.0116	0.6946

		151	Acetonitrile	0.0040	0.0023	-0.0016	0.0007	0.0122	0.0014	-0.0006	0.0115	0.6937
		NA	Chloroform	NA	NA	NA	NA	NA	NA	NA	NA	NA
		151	Dichloromethane	0.0039	0.0023	-0.0016	0.0007	0.0122	0.0016	-0.0006	0.0113	0.6893
		155	Methanol	0.0043	0.0025	-0.0017	0.0008	0.0130	0.0021	-0.0006	0.0115	0.6985
12	69(O) - 55(H)	161	Vacuum	0.0038	0.0029	-0.0020	0.0009	0.0150	0.0146	0.0033	-0.0029	0.7017
		157	Acetone	0.0012	0.0009	-0.0005	0.0003	0.0047	0.0042	0.0011	-0.0006	0.6199
		NA	Acetonitrile	NA	NA	NA	NA	NA	NA	NA	NA	NA
		NA	Chloroform	NA	NA	NA	NA	NA	NA	NA	NA	NA
		NA	Dichloromethane	NA	NA	NA	NA	NA	NA	NA	NA	NA
		159	Methanol	0.0019	0.0014	-0.0009	0.0005	0.0074	0.0068	0.0018	-0.0012	0.6658

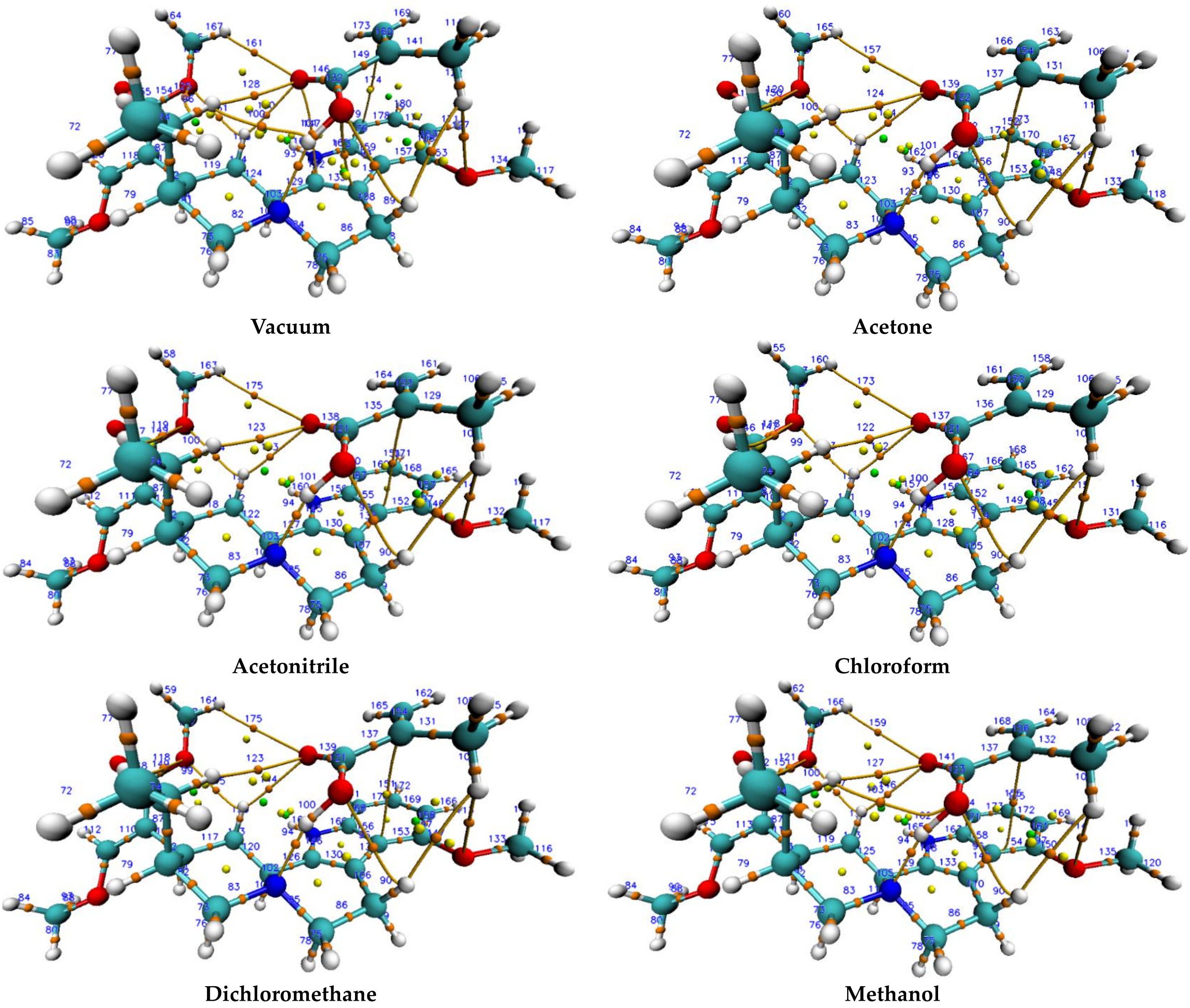

Figure S1. Bond critical point of complex 1 in all conditions

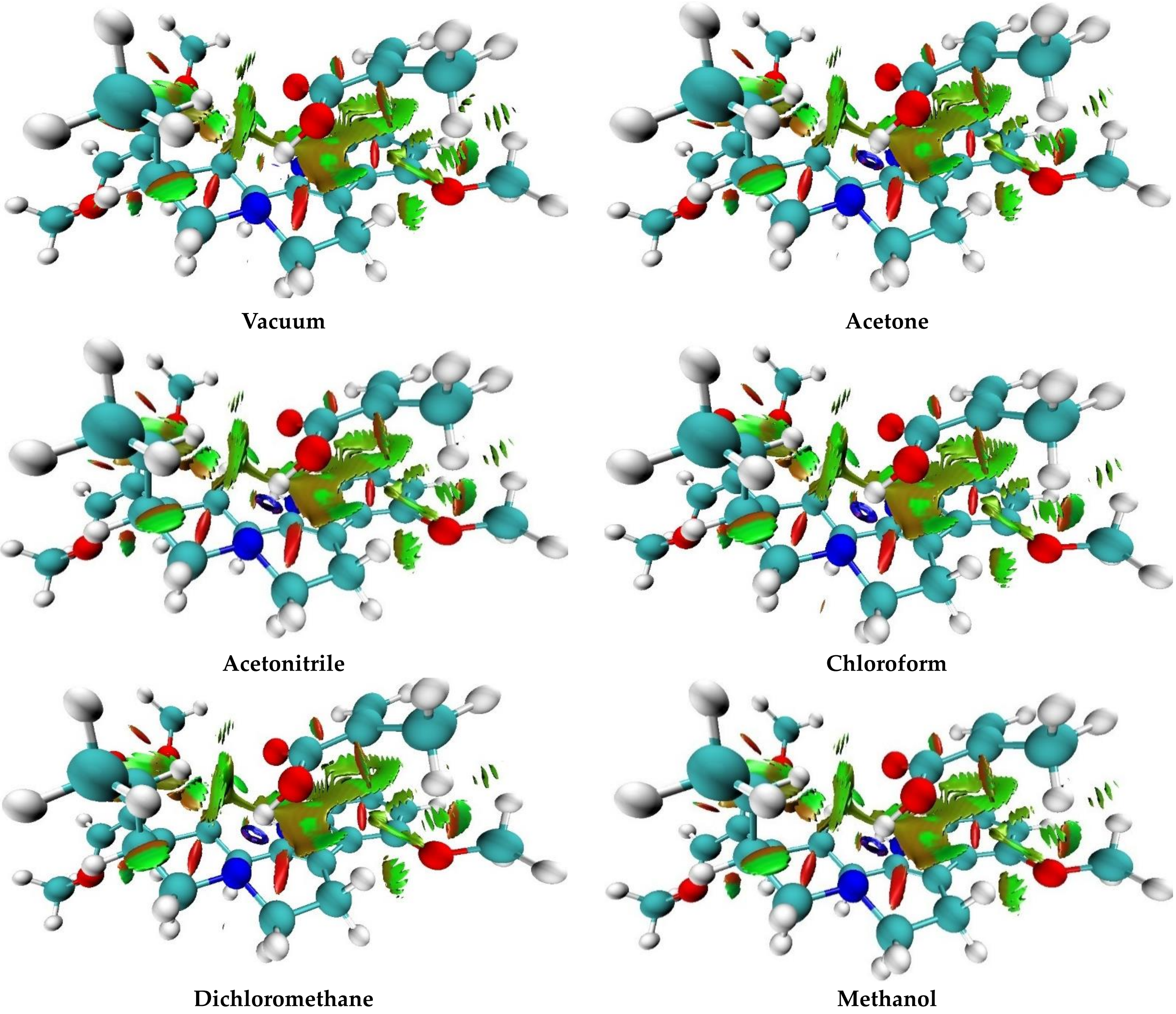

Figure S2. RDG isosurface of complex 1 in all conditions

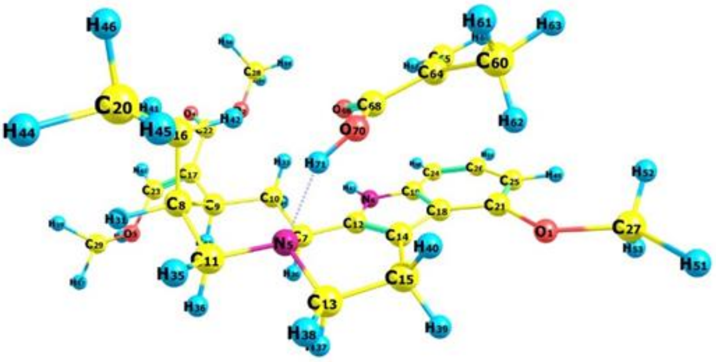

1-1

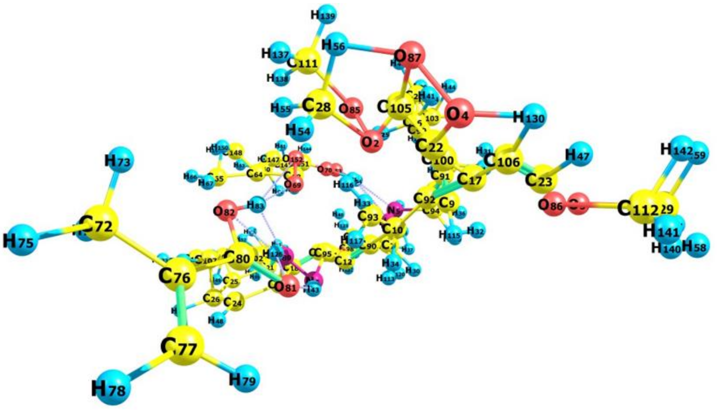

1-2

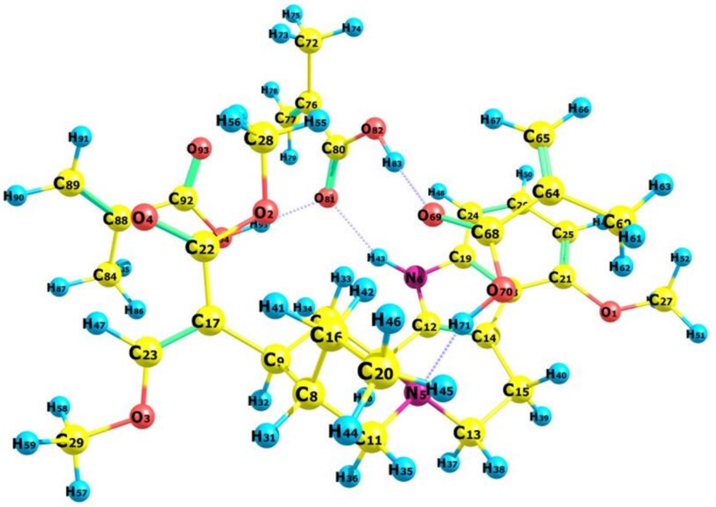

1-3

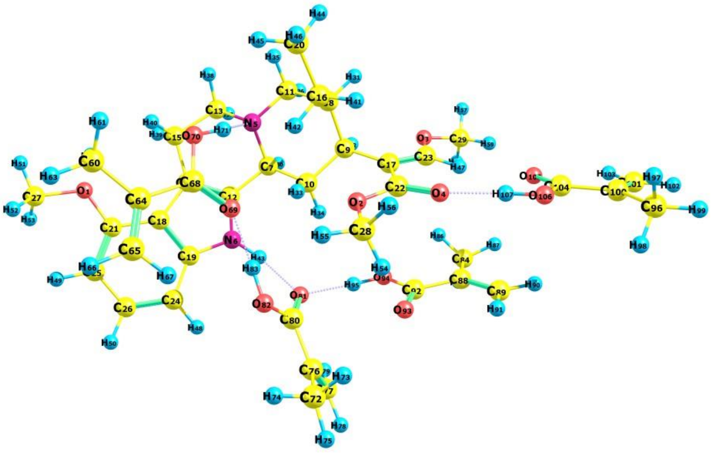

1-4

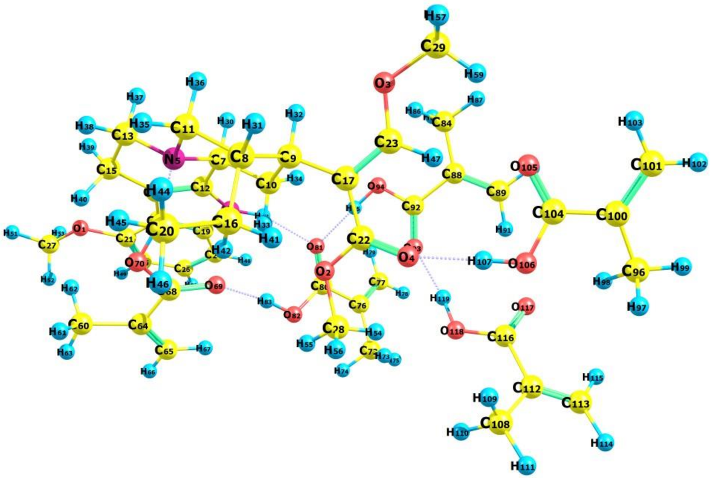

1-5

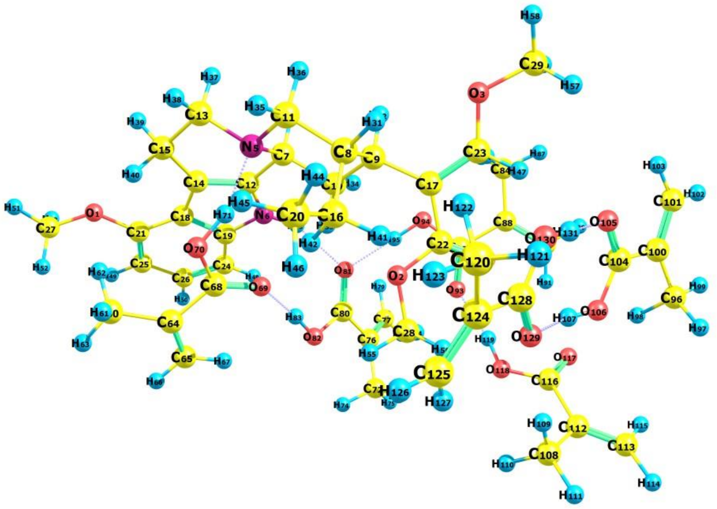

1-6

Figure S3. Binding mode of mitragynine with multi-monomer MAA

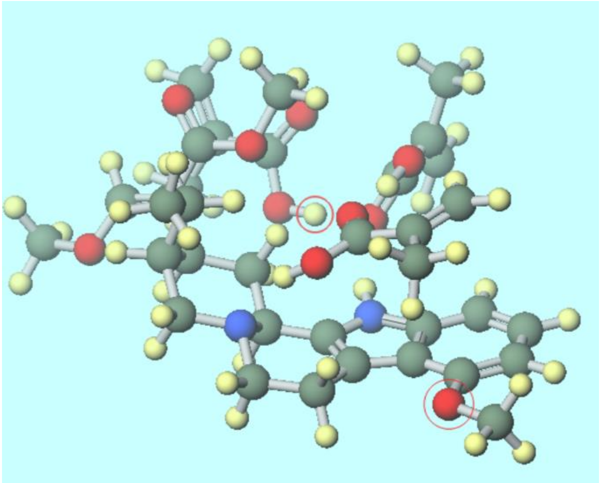

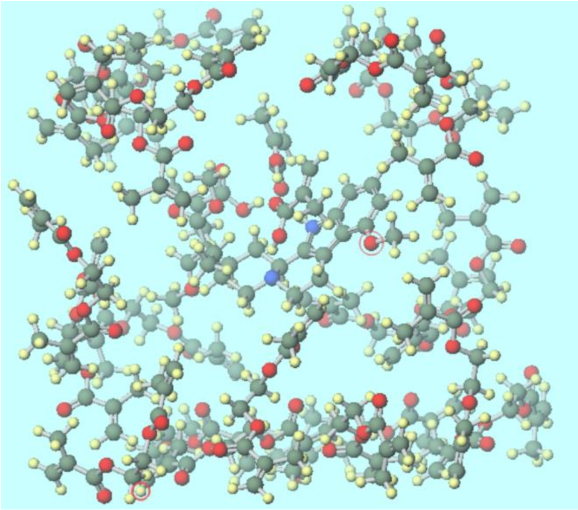

a b

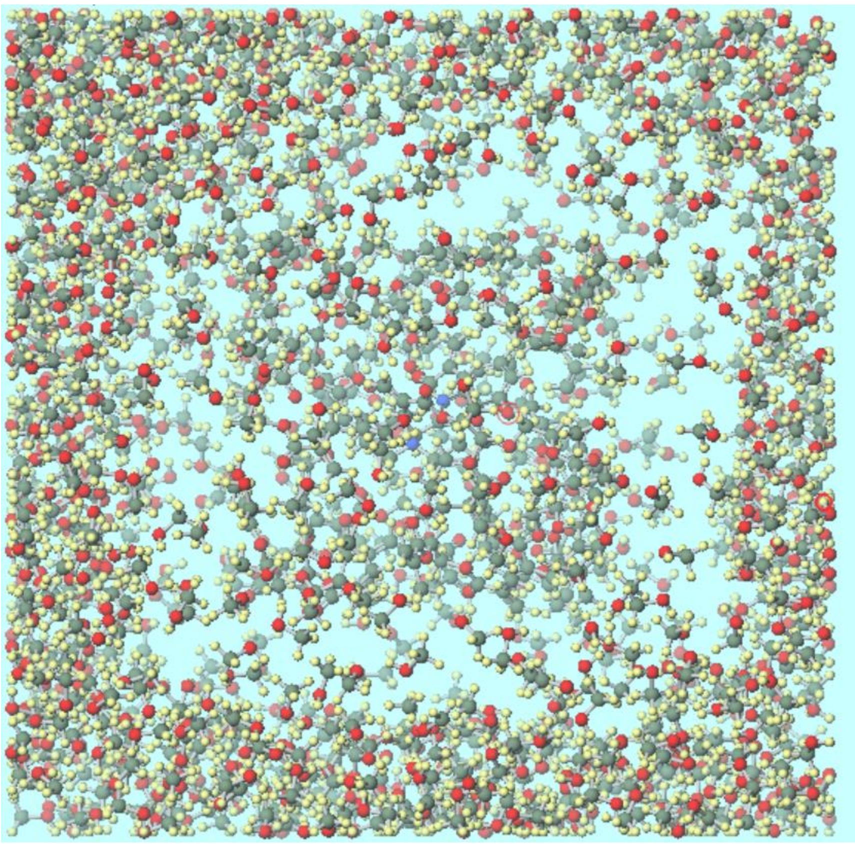

c

Figure S4. Molecular dynamic packing system: T-FM (a), T-FM-CL (b), and T-FM-CL-Solvent (c)

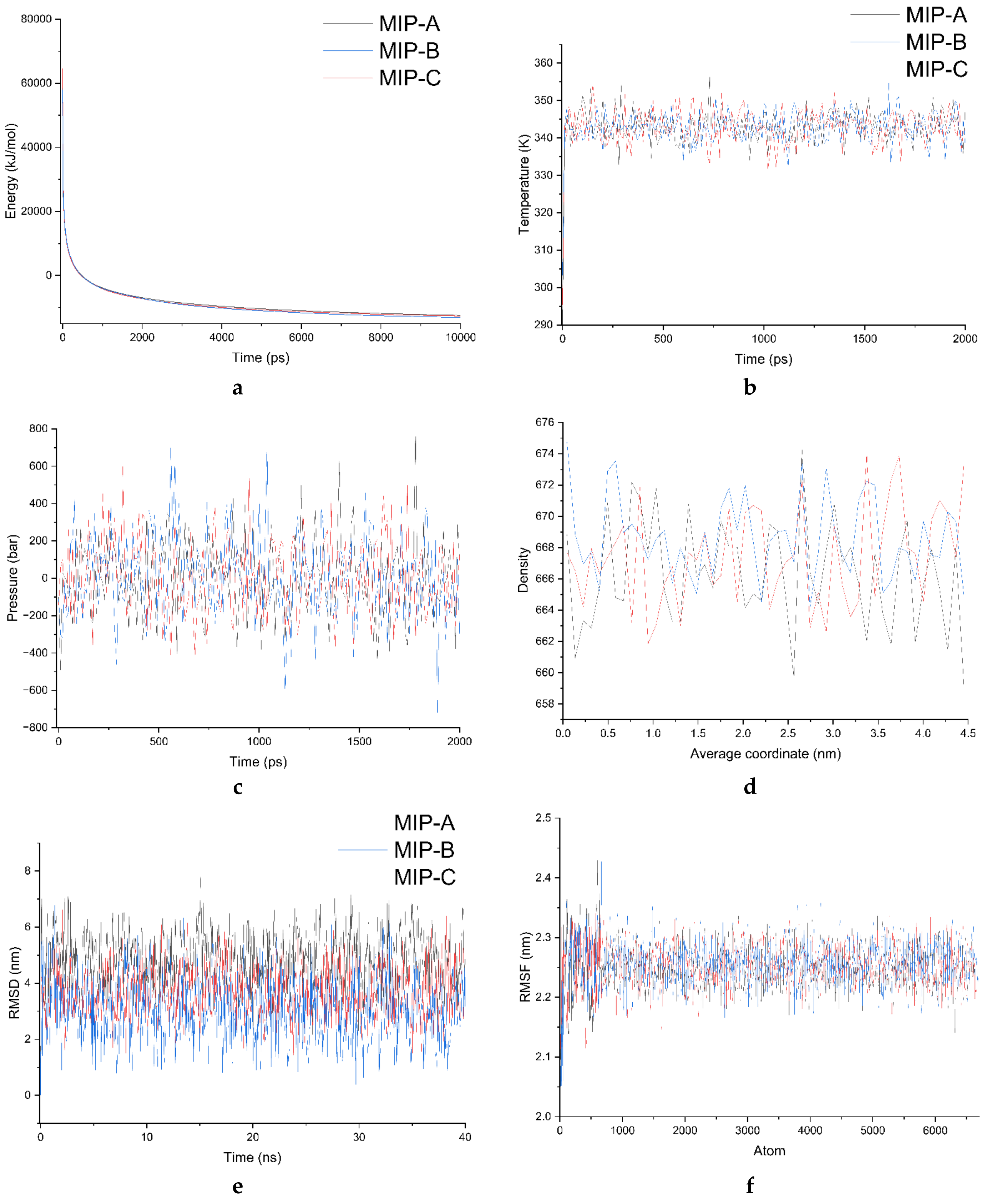

Figure S5. Molecular dynamics analysis: EM (a), NVT (b), NPT (c), density (d), RMSD (e), RMSF (f)
